# Advances in Natural Product Extraction: Established and Emerging Technologies

**DOI:** 10.3390/molecules31071136

**Published:** 2026-03-30

**Authors:** Carsyn R. Travis, Jared McMaster, Fatima Rivas

**Affiliations:** Department of Chemistry, Louisiana State University, 232 Choppin Hall, Baton Rouge, LA 70803, USA; ctrav18@lsu.edu (C.R.T.);

**Keywords:** natural products, extraction, bioactivity

## Abstract

Natural product research has experienced substantial growth over the past two decades, driven by a renewed appreciation for the structural complexity and biological relevance of compounds derived from nature. Technological advances in separation science, spectroscopic characterization, and high-sensitivity bioassays have collectively restored natural products to a position of prominence in modern drug discovery efforts. Nature remains the most prolific source of bioactive molecular diversity, drawing from microorganisms, plants, and marine life to offer a vast reservoir of structurally novel scaffolds whose pharmacological potential remains largely unexplored. Effective extraction and isolation remain foundational to natural product research, as the quality and purity of isolated compounds directly govern the reliability of downstream biological evaluation. Recent years have witnessed remarkable innovation in this space, spanning green and designer solvent systems, pressurized and ultrasound-assisted extraction platforms, supercritical fluid techniques, and integrated purification workflows that dramatically reduce processing time while improving compound recovery and analytical throughput. Particularly noteworthy is the growing application of artificial intelligence and machine learning tools for solvent selection, extraction optimization, and metabolite dereplication, which in combination with advanced phase-separation strategies and informatic platforms have substantially expanded the scope of detectable and characterizable metabolites within complex biological matrices. This review summarizes recent progress in extraction and isolation methodologies supporting natural product research, with particular emphasis on combinatorial extraction strategies, next-generation solvent systems, and AI-driven applications that have collectively improved operational efficiency, selectivity, and analytical output over the past five years.

## 1. Introduction

Natural products remain one of the most enduring and productive sources of structurally diverse bioactive molecules in pharmaceutical, nutraceutical, and cosmeceutical development. Despite the rapid expansion of combinatorial chemistry and synthetic libraries, compounds derived from plants, microorganisms, and marine organisms continue to contribute to clinically approved therapeutics, particularly in oncology, infectious disease, and metabolic disorders [1,2]. The intrinsic structural complexity, stereochemical richness, and evolutionary refinement of secondary metabolites confer biological specificity that is often difficult to replicate through purely synthetic approaches. However, the accessibility of these molecules fundamentally depends on the efficiency of extraction methodologies capable of transferring target metabolites from complex biological matrices into defined and analytically controllable forms. An examination of the conventional extraction techniques that have historically enabled the isolation of natural products provides essential context for understanding the practical accessibility of metabolites.

Conventional extraction techniques continue to form the operational foundation of natural product research, providing reliable and widely accessible approaches for recovering metabolites from complex biological matrices. Methods such as maceration, digestion, percolation, reflux, Soxhlet extraction, and steam distillation rely primarily on solvent diffusion and equilibrium partitioning to transfer compounds from plant tissues into an extracting medium. Their procedural simplicity, scalability, and long-standing reproducibility have made them standard tools in both laboratory investigations and industrial phytochemical processing. Despite the emergence of more advanced technologies, these conventional approaches remain widely used for preliminary phytochemical screening and large-scale extract production, particularly when robust and well-established workflows are required [3].

Building upon these established approaches, modern extraction technologies increasingly focus on intensifying solvent–matrix interactions and accelerating mass transfer to improve recovery of structurally diverse natural products. Techniques such as pressurized liquid extraction, supercritical fluid extraction, ultrasound- and microwave-assisted extraction, pulsed electric field extraction, and enzyme-assisted extraction achieve this by introducing controlled thermal, pressure-driven, mechanical, or biochemical inputs that promote solvent penetration and facilitate release of intracellular or matrix-bound metabolites. These developments allow greater control over extraction environments while improving solvent efficiency and reducing processing times. As a result, modern extraction strategies expand the range of accessible metabolite classes while supporting more efficient and sustainable workflows for natural product isolation. Hybrid approaches that incorporate mechanical disruption further enhance these processes by overcoming matrix resistance and enabling more complete metabolite recovery [4]. Beyond improvements in extraction methodology, increasing focus on integrating green solvents and computational tools to further refine natural product recovery are desirable.

Recent advances in natural product research increasingly integrate sustainable solvent engineering and computational optimization at the earliest stages of extraction and discovery. Green solvent systems have expanded the chemical space accessible through environmentally responsible extraction media, enabling improved solvation, selectivity, and preservation of sensitive metabolites. Machine learning and artificial intelligence frameworks can enhance extraction parameter optimization, dereplication, and prioritization by modeling nonlinear relationships between process variables and chemical outcomes. These advances lay the groundwork for downstream refinement, including advanced separation strategies and high-throughput analytical screening, collectively accelerating the identification of structurally diverse bioactive compounds. By integrating solvent innovation and predictive modeling prior to fractionation and screening, modern workflows reduce empirical trial-and-error experimentation while improving reproducibility, sustainability, and chemical resolution [5,6]. Within this developing field, a systematic overview of both established and emerging extraction strategies proves advantageous to contextualize their roles in modern natural product discovery.

This review covers advances in extraction methodology within the broader context of natural product research. First, major classes of natural products are outlined with emphasis on physicochemical characteristics that influence extraction behavior. Conventional extraction techniques are then evaluated, followed by discussion of modern technologies and hybrid systems. Finally, key considerations in separation, purification, and biological activity assessment are examined to contextualize extraction within the complete discovery pipeline. By integrating methodological principles with representative applications and recent technological developments, this review aims to provide a cohesive framework for selecting, optimizing, and implementing extraction strategies that support efficient and sustainable natural product discovery.

## 2. Natural Product Classes

Natural products have been central to human health for millennia, forming the chemical basis of Traditional Chinese Medicine, Ayurveda, and numerous Indigenous medical systems across the world. Crude preparations from plants, fungi, and other biological sources were historically used to treat acute and chronic diseases before their active constituents were chemically identified. Their enduring use reflects both their pharmacological relevance and their continued importance as a source of structurally diverse therapeutic agents in modern drug discovery [7].

Natural products are organic compounds produced by living organisms, such as plants, animals, fungi, bacteria, and marine organisms, through biosynthetic pathways. The classes of natural products include terpenes, alkaloids, phenolics, polyketides, and nonribosomal peptides. These compounds are considered secondary metabolites, or compounds not directly involved in the organism’s growth, development, or reproduction, but instead often serve ecological roles like defense, communication, or competition [8,9]. Variations in molecular architecture, functional composition, and physicochemical behavior contribute to the distinct chemical nature of these metabolite families, which fundamentally governs how they are accessed, manipulated, and interrogated in drug research [10].

### 2.1. Terpenes

**Figure 1:**
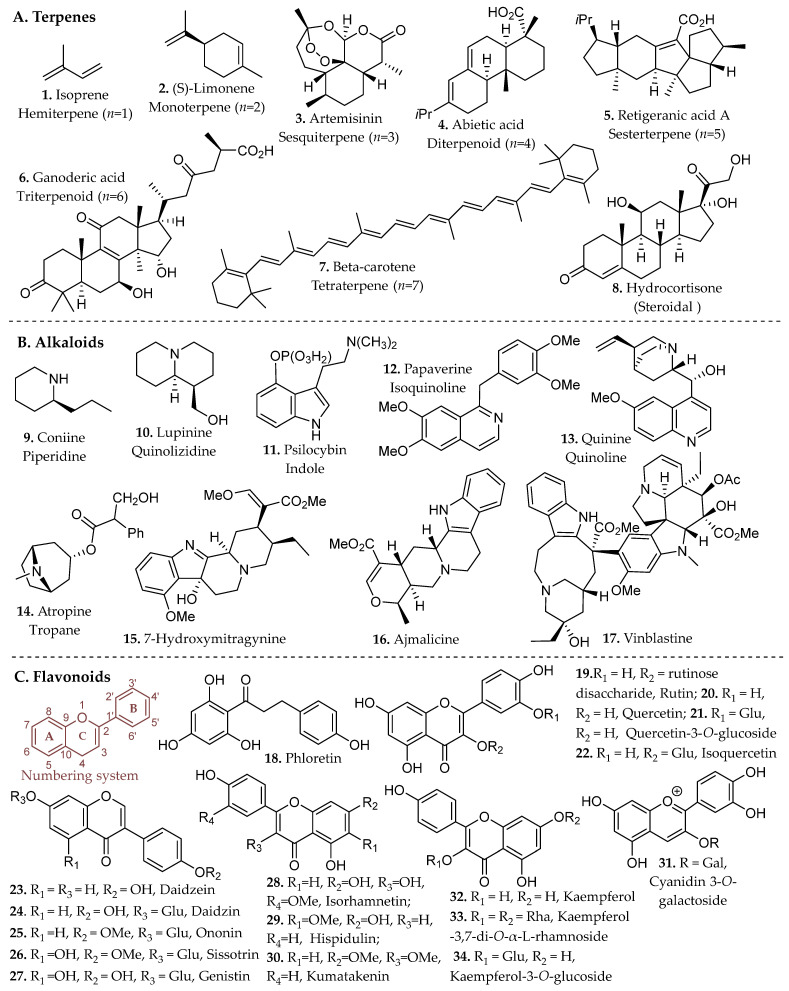
Representative natural products from three major classes: (**A**) Terpenes **1**–**8**, (**B**) Alkaloids **9**–**17**, and (**C**) Flavonoids **18**–**34**.

Terpenes constitute the largest and most structurally diverse class of natural products. They are biosynthetically derived from isoprene units (**1**, Figure 1) assembled through the mevalonate or methylerythritol phosphate pathways. Sequential condensation of these units produces linear prenyl intermediates that undergo cyclization and oxidative modification to generate monoterpenes, sesquiterpenes, diterpenes, triterpenes, and higher polyterpenes (**2**–**8**, Figure 1) [11].

The biological precursors are the activated C_5_ diphosphate intermediates, isopentenyl pyrophosphate (IPP) and its allylic isomer dimethylallyl pyrophosphate (DMAPP), generated via either the mevalonate (MVA) pathway in the cytosol or the methylerythritol phosphate (MEP) pathway in plastids. Sequential head-to-tail condensation of these units by prenyltransferases yields linear prenyl diphosphate intermediates of increasing chain length, including geranyl pyrophosphate (GPP, C_10_), farnesyl pyrophosphate (FPP, C_15_), and geranylgeranyl pyrophosphate (GGPP, C_20_), which serve as the direct precursors to monoterpenes, sesquiterpenes, and diterpenes, respectively [11,12]. Limonene (C_10_, **2**, Figure 1), a representative monoterpene, exemplifies the simplest cyclization product derived from GPP (*n* = 2 isoprene units), where terpene synthases catalyze carbocation-driven cyclization to generate the characteristic *p*-menthane skeleton. Additional tailoring reactions frequently introduce hydroxyl, carbonyl, or epoxide groups, converting hydrocarbon terpenes into more polar terpenoids [12].

The extraction behavior of terpenes is primarily dictated by molecular size and degree of functionalization. Nonpolar monoterpenes (C_10_) and sesquiterpenes (C_15_) lacking oxygenated groups are highly hydrophobic and are typically recovered using nonpolar solvents such as *n*-hexane, petroleum ether, or supercritical CO_2_. In contrast, oxygenated terpenoids exhibit increased polarity and hydrogen-bonding capacity. These compounds are more efficiently extracted using moderately polar solvents, including ethyl acetate, dichloromethane, methanol, or ethanol. Glycosylated terpenoids represent the most polar members of this class. The presence of sugar residues substantially increases aqueous solubility and promotes extraction into hydroalcoholic or water-rich systems.

Temperature and pressure also influence terpene recovery. Moderate heating enhances mass transfer and improves extraction efficiency for larger or moderately functionalized terpenoids. In supercritical fluid extraction, pressure adjustments above the critical point of CO_2_ increase solvent density and solvating strength. This allows selective tuning of terpene recovery without changing solvent identity. Accordingly, effective extraction strategies must be aligned with the polarity and thermal stability of the target terpene subclass [13].

Terpenes are predominantly derived from higher plants, where they accumulate in resin ducts, glandular trichomes, and essential oil glands. Conifers (*Pinus* spp.) and aromatic families such as Lamiaceae (*Salvia*, *Thymus*, *Rosmarinus*) and Myrtaceae (*Melaleuca alternifolia*) are major sources of bioactive monoterpenes such as limonene (**2**, Figure 1) [14,15]. Citrus species provide commercially important limonene for food, fragrance, and solvent applications, while *Cannabis sativa* supplies diverse mono- and sesquiterpenes with documented anti-inflammatory and anticancer potential. Higher terpenes such as diterpenes (e.g., taxol from *Taxus* spp., tanshinones from *Salvia miltiorrhiza*) and triterpenes (e.g., saponins from *Centella asiatica*) underpin clinically relevant therapies in oncology, cardiovascular medicine, and wound healing [15]. Terpenes constitute a structurally diverse class of secondary metabolites with far-reaching applications across multiple disciplines, including food preservation, agriculture, fragrance chemistry, and modern therapeutics, underscoring their significance as a rich and versatile chemical reservoir in both industrial and biomedical contexts.

### 2.2. Alkaloids

Alkaloids represent a vast array of architecturally complex class of secondary metabolites characterized by the presence of one or more nitrogen atoms, typically incorporated within heterocyclic frameworks. They are primarily derived from amino acid precursors such as tryptophan, tyrosine, ornithine, and lysine through enzyme-mediated transformations that generate indole, isoquinoline, quinoline, tropane, purine, pyrrolidine, and piperidine scaffolds (**9**–**17**, Figure 1) [16]. Recent advances in next-generation sequencing, genomics, metabolomics, and bioinformatics have enabled the emergence of synthetic biology as a promising strategy to address supply limitations for these compounds. The biosynthesis of alkaloids in bacterial and yeast hosts has demonstrated considerable success in improving yields of these natural products [17].

A defining feature of alkaloids is the basicity of the nitrogen atom. The protonation state of this functional group strongly influences aqueous solubility and solvent partitioning behavior. Under acidic conditions (pH < pK_a_), alkaloids are converted to protonated, water-soluble salts. Under basic conditions (pH > pK_a_), they revert to neutral free bases that exhibit greater affinity for organic solvents such as chloroform, dichloromethane, or ethyl acetate. This reversible acid–base behavior forms the basis of classical liquid–liquid extraction strategies.

In practice, alkaloid extraction relies on acid-base partitioning: initial acidification transfers protonated alkaloids to the aqueous phase, after which alkalization facilitates back-extraction into an organic solvent. Controlled pH adjustment enables selective partitioning based on differences in basicity among alkaloid subclasses and facilitates purification through iterative extraction cycles [18].

As an illustrative example, 7-hydroxymitragynine (**15**, Figure 1), an alkaloid from *Mitragyna speciosa* is commonly isolated using acid–base extraction protocols that exploit the pH-dependent protonation equilibrium of its tertiary amine [19]. Treatment with diluted acid converts the alkaloid to a water-soluble ammonium salt, enabling selective transfer into the aqueous phase while neutral lipophilic constituents remain in the organic layer. Subsequent alkalization regenerates the neutral free base, which can then be partitioned into an organic solvent for concentration and purification. Because 7-hydroxymitragynine is typically present at low abundance in native plant material, acid–base enrichment can substantially increase its relative proportion within the final extract. Such extraction-driven enrichment can significantly alter the concentration of individual alkaloids within the final extract, underscoring the importance of pH control in modulating chemical composition and biological activity [20].

Industrial and medicinally relevant alkaloids are concentrated within specific botanical sources that continue to support pharmaceutical production. Alkaloids occur across diverse taxa including *Coffea* spp., *Erythroxylum coca*, and *Cinchona* spp., with well-established clinical derivatives emerging from these genera. Rubiaceae and related taxa provide quinoline alkaloids such as quinine (**13**, Figure 1) from *Cinchona officinalis*, historically foundational in antimalarial therapy and still pharmaceutically relevant. Solanaceae species, including *Atropa belladonna* and *Datura stramonium*, furnish tropane alkaloids such as atropine (**14**, Figure 1) and scopolamine used in anticholinergic and anesthetic applications [21]. Papaveraceae (*Papaver somniferum*) remains the primary industrial source of morphine and codeine, cornerstone opioid analgesics in modern medicine, while Apocynaceae (*Catharanthus roseus*) supplies vincristine and vinblastine (**17**, Figure 1), microtubule-disrupting agents essential to contemporary chemotherapy regimens [22]. These plant families represent scalable and chemically diverse reservoirs of nitrogen-containing scaffolds that continue to drive drug discovery, semi-synthesis, and industrial alkaloid production.

### 2.3. Flavonoids and Related Compounds

**Figure 2:**
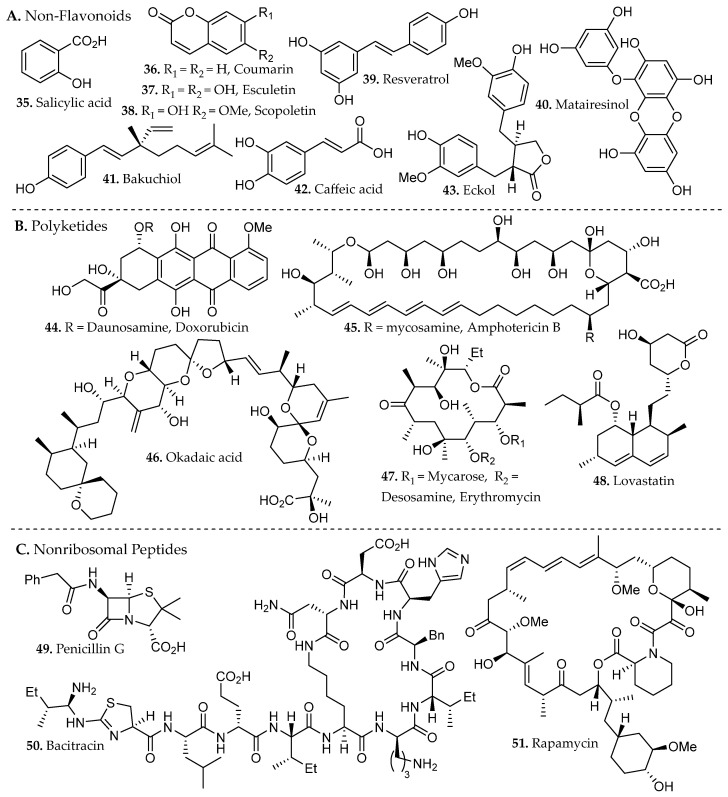
Representative natural products from three major classes: (**A**) Non-flavonoids **35**–**43**, (**B**) Polyketides **44**–**48**, and (**C**) Nonribosomal peptides **49**–**51**.

Phenolic compounds arise primarily from the shikimate and aceto-malonate pathways, which intersect through the phenylpropanoid network. The shikimate pathway generates aromatic amino acids that give rise to simple phenolic acids through the phenylpropanoid network [23]. Flavonoids are subsequently formed through enzymatic coupling of phenylpropanoid intermediates with malonyl-derived units from the aceto-malonate pathway, thereby establishing the core flavonoid scaffold. Further enzymatic tailoring yields diverse flavonoid subclasses, while additional oligomerization or polymerization reactions generate condensed tannins and related polyphenolic structures from flavan-3-ol precursors [24]. Overall, these biosynthetic pathways yield several major subclasses of phenolic compounds, including flavonoids (**18**–**34**, Figure 1), and non-flavonoids such as phenolic acids, coumarins, stilbenes, tannins, and lignans (**35**–**43**, Figure 2).

Flavonoids consist of two aromatic rings (A and B, Figure 1C) linked by a three-carbon bridge that forms a heterocyclic C ring, generating a 2-phenylchroman scaffold. Structural substitution strongly influences solubility and, consequently, extraction behavior. Simple phenolic acids, such as salicylic and caffeic acid (**35**, **42**, Figure 2), exhibit relatively high aqueous solubility due to low molecular weight and ionizable carboxylic acid groups (pK_a_ ~4–5), which promote pH-dependent formation of water-soluble carboxylate species [23].

In contrast, flavonoids possess larger, π-conjugated polycyclic systems with greater hydrophobic surface area. Although multiple hydroxyl groups permit hydrogen bonding, aglycone forms generally display limited intrinsic aqueous solubility due to molecular rigidity and aromatic character [25]. Glycosylation at positions such as C-3 or C-7 as numbered in Figure 1C, increases polarity, hydrogen-bonding capacity, and overall solubility relative to the parent aglycone, directly influencing solvent compatibility and extraction strategy [25].

Accordingly, phenolic acids and glycosylated flavonoids are efficiently extracted using polar solvents such as water, methanol, or ethanol, and in some cases, under mildly acidic conditions [26]. Aglycone and methylated flavonoids are commonly extracted with mixed solvent systems (e.g., methanol–water, ethanol–water, or acetone–water) to balance solubilization of aromatic cores and polar functionalities. While elevated temperature enhances solubility and mass transfer, thermal sensitivity necessitates optimization. Sequential extraction using solvents of increasing polarity (e.g., hexane followed by ethyl acetate or methanol) enables polarity-based fractionation, simplifying purification and targeted isolation.

Phenolic compounds of industrial and medicinal relevance are concentrated in specific botanical taxa and agro-industrial streams. Hydroxycinnamic acids (e.g., caffeic and ferulic acids), flavanols such as quercetin and kaempferol, and anthocyanins are widely distributed across cereals, legumes, citrus fruits, berries, and *Camellia sinensis* (tea tree), directly linking these matrices to antioxidant, anti-inflammatory, and cardioprotective applications [27].

At industrial scale, lignin derived from softwoods and hardwoods (e.g., coniferous species and *Populus* spp.), condensed tannins from bark and heartwood, and cashew nutshell liquid from *Anacardium occidentale* constitute high-tonnage phenolic streams already integrated into resin, adhesive, and polymer manufacturing [27]. Agro-industrial by-products such as onion peels (*Allium cepa*), tomato pomace (*Solanum lycopersicum*), and pistachio green hulls (*Pistacia vera*) have been identified as concentrated reservoirs of quercetin derivatives, rutin (**19**, Figure 1), protocatechuic acid (**53**, Figure 4), and related phenolics, reinforcing their relevance as scalable feedstocks for metabolite recovery [28]. Collectively, these plant matrices and processing residues represent chemically diverse and industrially viable sources of phenolic scaffolds for pharmaceutical, nutraceutical, and materials-oriented applications.

### 2.4. Polyketides

Polyketides (**44**–**48**, Figure 2) are a major class of secondary metabolites derived from the acetate pathway. Their assembly is mechanistically analogous to fatty acid biosynthesis and is catalyzed by polyketide synthases (PKSs), which extend an acyl starter unit through iterative decarboxylative Claisen condensations with malonyl-derived C_2_ or C_3_ building blocks tethered to coenzyme A or an acyl carrier protein [29].

Structural diversity arises from the variable processing of the β-keto intermediates formed during chain elongation. Unlike fatty acid synthases, which fully reduce each elongation product, PKSs selectively control reduction and modification steps, thereby generating backbones that retain ketone, alcohol, or olefin functionalities at defined positions. This flexible control of oxidation state, combined with variation in extender units and post-assembly tailoring reactions, produces the extensive structural diversity characteristic of polyketide natural products [29].

Polyketides are classified by their structure and oxidation pattern into several subclasses, namely linear and cyclic fatty acid-like polyketides, aromatic polyphenols (anthraquinones, tetracyclines), and polypropionates [30]. Polypropionates include macrolide antibiotics, linear polyketides, and polyethers. Polyketide solubility varies widely depending on the balance between hydrophobic carbon frameworks and polar groups (carbonyls, hydroxyls, lactones). Less functionalized and macrocyclic polyketides dissolve preferentially in aprotic polar solvents (ethyl acetate, dichloromethane), while polyhydroxylated aromatic polyketides and quinones show greater solubility in protic polar solvents (methanol, aqueous alcohols). Mixed solvent systems should be considered to achieve efficient recovery, particularly when targeting glycosylated or highly conjugated polyketide metabolites embedded within lignocellulosic matrices.

### 2.5. Nonribosomal Peptides

Nonribosomal peptides (NRPs) (**49**–**51**, Figure 2) are structurally multifaceted secondary metabolites synthesized by multimodular nonribosomal peptide synthetases (NRPSs), primarily in bacteria and fungi. NRPSs function as molecular assembly lines that sequentially activate, attach, and link amino acid building blocks, including unusual amino acids not found in standard proteins such as *D*-amino acids, *N*-methylated amino acids, and heterocyclic units. Additional modification steps, including epimerization, methylation, oxidation, cyclization, further increase structural diversity before the final peptide product is released through enzyme-catalyzed cyclization or cleavage [31]. This biosynthetic process yields linear, cyclic, and macrocyclic peptides with varied functionalization and specific stereochemistry, which contributes to NRP bioactivity.

NRPs are commonly acquired through solvent extraction of microbial biomass or culture supernatants, with solvent selection guided by peptide polarity, and degree of lipidation. Moderately polar organic solvents such as methanol, *n*-butanol, or aqueous alcohol mixtures efficiently recover many NRPs, while more lipophilic lipopeptides often require the use of aprotic polar solvents (ethyl acetate or chloroform), biphasic extraction, or solid-phase adsorption strategies. Buffer solution can also be used to limit the risk of protein aggregation. The modular architecture of NRPS systems, coupled with extensive post-assembly tailoring, underpins both the structural diversity of NRPs and the need for extraction strategies tailored to peptide charge state, amphiphilicity, and stability [32].

### 2.6. Natural Products in Medicine

Natural products have historically served as one of the most productive sources of therapeutic agents and continue to provide structurally diverse scaffolds for modern drug discovery. Across multiple therapeutic areas, including infectious disease, oncology, and metabolic disorders, natural product scaffolds have contributed substantially to clinically approved drugs and preclinical candidates. Natural product and natural-product-derived compounds show a steady enrichment from Phase I to Phase III rising from ~35% in Phase I to ~45% in Phase III while synthetic compounds show the inverse attrition trend (decrease from ~65% to ~55%). Natural products and their derivatives were also found to be less toxic than synthetic counterparts in in vitro and in silico studies [33].

The annual approval rate of natural product-derived molecules has fluctuated between 0 and 8, averaging approximately five approvals per year from 2014 through 2024. Despite this steady pipeline contribution, the pace of genuinely novel pharmacophore discovery has slowed considerably. Of the 33 new pharmacophores not previously represented in approved drugs that are currently in clinical development, only one has been identified in the past 15 years [34]. This trend underscores both the enduring therapeutic value of natural product scaffolds and the growing need for advanced discovery strategies capable of unlocking the chemical novelty that remains embedded within underexplored biological sources.

Natural products occupy broader chemical space than synthetic libraries, with fused ring systems, dense stereocenters, and functionalized carbon frameworks that confer precise three-dimensional fit with protein binding sites and allosteric pockets. This structural complexity makes them uniquely suited for modulating challenging targets such as protein–protein interactions and intrinsically disordered proteins, positioning natural product scaffolds as privileged templates for next-generation drug discovery [35,36].

Several representative natural products illustrate the chemical diversity and therapeutic impact of these scaffolds. Artemisinin (**3**, Figure 1), a sesquiterpene endoperoxide isolated from *Artemisia annua*, revolutionized malaria treatment and remains a cornerstone of modern antimalarial therapy. Its characteristic endoperoxide bridge undergoes Fe(II)-mediated activation within the malaria parasite, generating reactive radical species that damage essential parasite proteins and lipids and ultimately cause rapid parasite death [37,38]. In contrast, the quinoline alkaloid quinine (**13**, Figure 1), isolated from *Cinchona* bark, represents the historical precedent for contemporary antimalarial drug development. Quinine operates through a mechanistically distinct pathway, interfering with parasitic heme detoxification and promoting the accumulation of toxic hemin complexes that contribute to parasite death through oxidative stress and inhibition of cysteine proteases [38].

Natural products have also played a central role in cancer chemotherapy. The vinca alkaloid vinblastine (**17**, Figure 1), obtained from *Catharanthus roseus*, remains an important frontline chemotherapeutic agent used in the treatment of hematological malignancies, including Hodgkin lymphoma and non-Hodgkin lymphoma. Industrial production relies on semi-synthetic coupling of the monomeric alkaloids catharanthine and vindoline (unit I and unit II, respectively, of **17**, Figure 1) [39], highlighting the continued importance of plant-derived intermediates in pharmaceutical manufacturing. Similarly, microbial natural products have produced numerous antibiotics and anticancer agents, exemplified by erythromycin (**47**, Figure 2), a macrolide polyketide assembled by modular biosynthetic pathways that inhibits bacterial protein synthesis through interactions within the 50S ribosomal exit tunnel [40].

Beyond these clinically established drugs, naturally occurring scaffolds continue to influence contemporary biomedical research. The flavonoid quercetin (**20**, Figure 1), widely distributed in fruits and vegetables, has been extensively studied for its antioxidant and anti-inflammatory properties and its ability to modulate cellular signaling pathways involved in apoptosis, autophagy, and metabolic regulation [41]. Likewise, bakuchiol (**41**, Figure 2), a prenylated phenolic meroterpenoid isolated from *Psoralea corylifolia*, exhibits retinoid-like biological activity through regulation of gene networks associated with collagen synthesis, extracellular-matrix remodeling, and inflammatory control [42]. Together, these examples illustrate how natural products continue to provide chemically diverse scaffolds that inspire new therapeutic strategies and guide modern medicinal chemistry efforts. Accessing these potentially bioactive compounds requires efficient extraction methods capable of freeing metabolites from complex matrices.

## 3. Conventional Extraction Methods

The structural diversity of secondary metabolites continues to drive innovation in medicinal chemistry. Extraction of these bioactive compounds represents a critical first step in natural product research, providing the foundation for downstream purification, structural characterization, and pharmacological evaluation. Extraction is the initial step in accessing these compounds from biological matrices, requiring conditions that account for solubility, polarity, and mass-transfer behavior to transfer metabolites from complex matrices into defined solvent phases. Key parameters such as solvent-to-solid ratio, temperature, and extraction time govern both yield and chemical stability, making careful control essential for preserving labile functional groups and representative metabolite profiles [43]. Because secondary metabolites span wide ranges of polarity, molecular weight, and reactivity, extraction strategies must be aligned with the physicochemical properties of each class to ensure accurate characterization and meaningful biological evaluation.

Conventional extraction methods such as maceration, digestion, decoction, percolation, reflux extraction, Soxhlet extraction, and steam distillation have historically served as foundational techniques in phytochemical isolation. These approaches offer broad accessibility, cost-effectiveness, and adaptability across diverse plant matrices. They typically operate at atmospheric pressure with extended contact times and substantial solvent volumes, conditions that favor complete tissue penetration but may present challenges for thermally sensitive analytes or require extended processing durations.

### 3.1. Maceration

**Figure 3:**
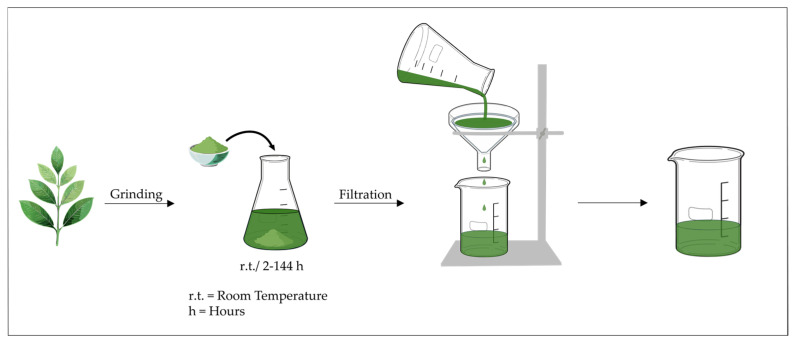
Maceration extraction scheme.

Maceration involves submerging comminuted plant material in a suitable solvent at room temperature (r.t.) or mildly elevated temperatures, allowing metabolites to diffuse from the matrix over time (Figure 3). Extraction is commonly carried out in glass or stainless-steel vessels with periodic agitation or stirring to enhance mass transfer. Hydroalcoholic systems are frequently used, with reported solid-to-solvent ratios of approximately 1:10–1:30 (g:mL) and extraction times ranging from several hours (h) to days. After filtration to remove residual solids, the solvent is evaporated under reduced pressure to yield the crude extract [44]. Maceration is widely used for preliminary phytochemical screening and standardized botanical preparations due to its operational simplicity and compatibility with thermolabile compounds. However, it is a diffusion-limited process and is often slower or less efficient than intensified techniques such as ultrasound- or microwave-assisted extraction.

**Figure 4:**
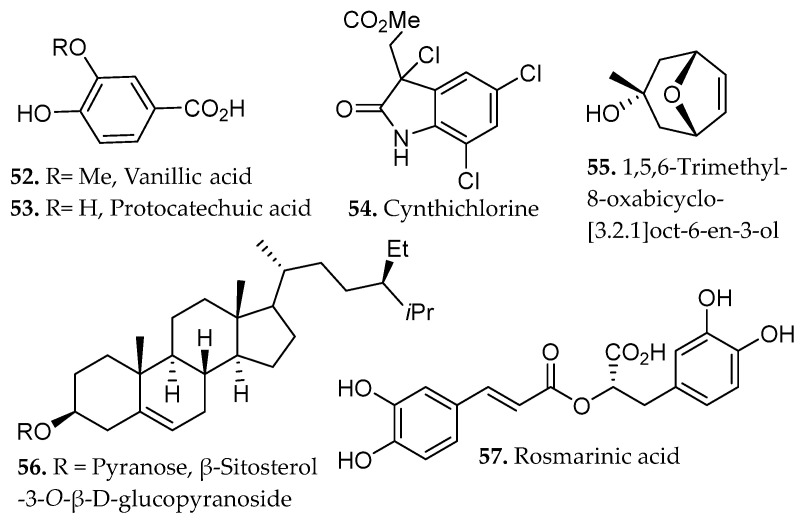
Natural products **52**–**57** extracted by conventional maceration.

Maceration of *Mentha longifolia* (70% ethanol, 72 h, r.t., no external heating) produced the highest extraction yield among the tested methods, yielding approximately 2.3-fold greater recovery than ultrasound-assisted extraction (UAE) and nearly threefold greater recovery than Soxhlet extraction under identical solvent conditions. The method enabled efficient recovery of phenolic acids such as vanillic, protocatechuic, and rosmarinic acids (**52**–**53**, **57**, Figure 4), as well as flavonoids such as rutin, quercetin, and kaempferol (**19**–**20**, and **32**, respectively Figure 1) [45].

Comparative evaluation against Soxhlet and UAE demonstrated that both maceration and Soxhlet extraction with 70% ethanol preserved oxidation sensitive phenolics more effectively than UAE, yielding extracts with superior antioxidant, antiradical, and antimicrobial activity against *S. aureus* and *E. coli*. The absence of elevated temperature and mechanical disruption in maceration likely contributed to improved retention of thermolabile and oxidation-prone metabolites [45].

Optimization of maceration for the marine ascidian *Cynthia savignyi* has enabled efficient recovery of the thermolabile β-carboline alkaloid cynthichlorine (**54**, Figure 4) under mild extraction conditions [46]. Optimal maceration conditions of 25 °C, 12 h, and a 75% ethanol/25% water solvent system were established using a Box–Behnken response-surface design, a statistical optimization method that evaluates nonlinear interactions among multiple extraction variables to identify maximal yield with minimal experimental runs. Under these conditions, cynthichlorine recovery increased by more than twofold relative to Soxhlet extraction. The optimized macerate retained strong cytotoxic activity against glioblastoma, breast, and prostate cancer cell lines, suggesting that ambient-temperature maceration effectively preserves the thermosensitive marine metabolites.

Sequential maceration of *Siparuna cymosa* using solvents of increasing polarity (*n*-hexane, chloroform, ethyl acetate, and ethanol) successfully isolated 1,5,6-trimethyl-8-oxabicyclo[3.2.1]oct-6-en-3-ol, β-sitosterol-3-*O*-β-D-glucopyranoside (**55**–**56**, Figure 4), and kaempferol-3,7-di-*O*-α-L-rhamnoside (**33**, Figure 1) [47]. The kaempferol glycoside demonstrated selective cytotoxicity against leukemia cells with minimal effects on normal blood cells. The mild extraction conditions were credited for the preservation of labile glycosidic and phenolic moieties.

Recent methodological advances have evolved maceration from a passive diffusion technique into a sophisticated and controllable extraction platform. A notable innovation is the triphasic solvent system, which substitutes sequential polarity-gradient extractions with a self-partitioning solvent mixture that spontaneously generates distinct polar, intermediate, and nonpolar phases [48]. An optimized solvent system comprising *n*-heptane, ethyl acetate, acetonitrile, 1-butanol, and water in a ratio of 22:14:29:8:27 demonstrated superior extraction performance relative to conventional methods. This multi-solvent formulation reached liquid–liquid equilibrium within 8 h and produced higher extract yields compared to exhaustive maceration. Notably, it also achieved twofold greater yields than methanolic Soxhlet extraction, highlighting the advantages of a rationally designed polarity-gradient solvent system in maximizing the recovery of structurally prolific natural products.

**Figure 5:**
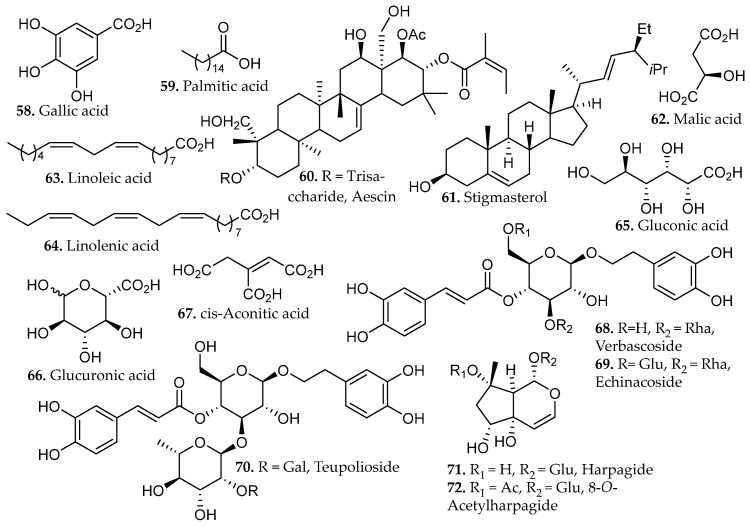
Natural products **58**–**72** extracted using a triphasic solvent maceration method.

Application of this solvent system to *Anthyllis vulneraria* demonstrated effective phase-selective partitioning of molecularly heterogeneous metabolites [48]. Notably, the carbohydrate glucose and the lipid palmitic acid (**59**, Figure 5) segregated into the polar and nonpolar phases, respectively, consistent with their contrasting physicochemical properties. Meanwhile, a broad range of phenolic and amphiphilic natural products, including gallic acid (**58**, Figure 5), caffeic acid (**42**, Figure 2), rutin (**19**, Figure 1), rosmarinic acid (**57**, Figure 4), quercetin (**20**, Figure 1), and the triterpenoid saponin aescin (**60**, Figure 5), preferentially concentrated in the intermediate layer.

This phase-selective distribution reflects the capacity of the multi-solvent system to simultaneously accommodate compounds spanning a wide range of polarities, underscoring its utility as a comprehensive extraction platform for complex plant matrices [48]. This approach substantially reduced solvent requirements and time required for extraction while facilitating targeted enrichment of bioactive constituents for downstream pharmacological evaluation.

In an independent study, *Ajuga pyramidalis* extracted under triphasic maceration achieved cumulative extraction exceeding 50% of dry plant mass [49]. The nonpolar solvent phase predominantly contained palmitic acid, stigmasterol, linoleic acid, and linolenic acid (**59**, **61**, **63**–**64**, Figure 5), while the polar solvent phase contained malic acid, gluconic acid, glucuronic acid, and *cis*-aconitic acid (**62**, **65**–**67**, Figure 5). The intermediate fraction exhibited selective enrichment of chemically heterogeneous compound classes, most notably phenylethanoid glycosides verbascoside, echinacoside, and teupoloside (**68**–**69**, Figure 5) and iridoids harpagide and 8-*O*-acetylharpagide (**67**–**68**, Figure 5). Several of these compounds represent new phytochemical contributions to the known chemical profile of *Anthyllis vulneraria*. Biological evaluation of the resultant extracts revealed that the polar fraction and iridoid-enriched fractions exhibited promising modulation of genes associated with epidermal renewal, highlighting their potential utility in cosmetic and dermatological applications. These findings highlight phase-selective extraction as a powerful strategy for comprehensive metabolite recovery and targeted isolation of functionally relevant fractions.

Technological advancement has further yielded Rapid Solid–Liquid Dynamic Extraction (RSLDE), a pressure-modulated iteration of conventional maceration wherein cyclic compression and decompression within the extraction vessel generates pressure gradients that actively facilitate solute migration from plant matrices [50].

**Figure 6:**
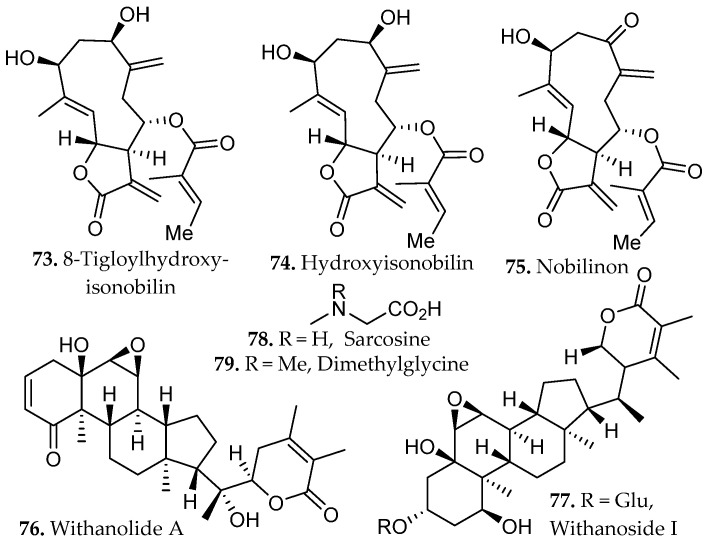
Natural products **73**–**79** extracted by rapid solid–liquid dynamic extraction.

RSLDE using the Naviglio^®^ apparatus demonstrated high efficiency for the recovery of metabolites from *Chamaemelum nobile* when compared with conventional maceration and UAE [50]. RSLDE performed using EtOH/H_2_O mixtures, particularly at 50% ethanol, provided extracts enriched in flavonoids, such as isorhamnetin, hispidulin, and kumatakenin (**28**–**30**, Figure 1), and sesquiterpenoids, such as 8-tigloylhydroxyisonobilin, hydroxyisonobilin, and nobilinon (**73**–**75**, Figure 6), as confirmed by UPLC-HRMS profiling. Multivariate statistical analysis (PCA) clearly separated RSLDE extracts from those obtained by maceration and UAE, indicating a distinct and more concentrated chemical fingerprint driven by the dynamic extraction mechanism. While UAE promoted partial release of metabolites, it did not achieve the same breadth or intensity of flavonoid enrichment observed with RSLDE. Importantly, the RSLDE approach combined reduced extraction time with mild thermal conditions and green solvent systems, preserving metabolite integrity while enhancing extraction kinetics.

An integrated metabolomic evaluation of *Withania somnifera* root extracts demonstrated that RSLDE provides a chemically rich extract profile relative to maceration and UAE under comparable solvent systems [51]. LC-ESI-HRMS analysis revealed that all extraction methods recovered the characteristic steroidal lactones of *W. somnifera*, such as withanolide A and withanoside I (**76**–**77**, Figure 6), yet multivariate analysis showed clear method-dependent clustering, with RSLDE extracts enriched in lower molecular-weight, semi-polar constituents. RSLDE favored the extraction of vanillic acid (**52**, Figure 4), sarcosine, dimethylglycine (**78**–**79**, Figure 6), benzoic acid, and tropane alkaloid compounds that were less prominent in maceration and ultrasonic extracts. The RSLDE extract using 100% methanol also exhibited the highest tyrosinase inhibitory activity. These findings therefore establish RSLDE as a versatile and chemically efficient extraction platform capable of selectively enriching chemically secondary metabolites while maintaining extract integrity under mild conditions, supporting its applicability as a sustainable alternative to conventional solid–liquid extraction strategies

These developments represent a meaningful evolution in maceration-based extraction. Triphasic solvent systems improve compound selectivity by exploiting differential polarity partitioning across solvent phases, while RSLDE enhances mass transfer through repeated pressure cycling. Importantly, both strategies preserve the scalability and operational accessibility characteristic of conventional maceration, while demonstrating measurable improvements in extraction efficiency and expanded utility for the isolation of bioactive natural products.

### 3.2. Digestion

**Figure 7:**
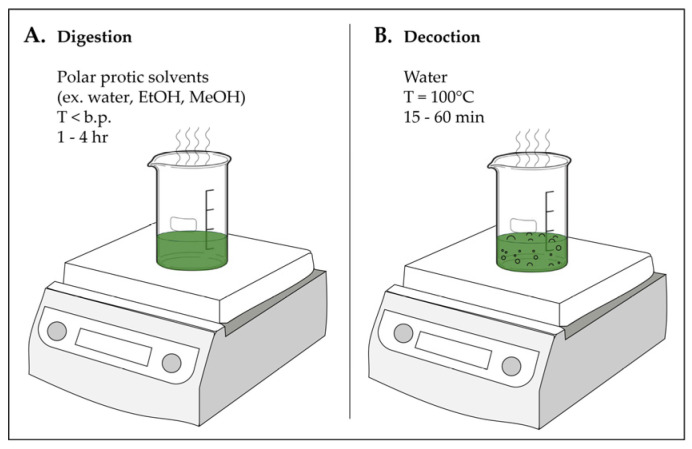
(**A**) Digestion and (**B**) decoction extraction diagrams.

Digestion enhances maceration by applying controlled sub-boiling heat (35–70 °C, 1–4 h), with optimal parameters dependent on the plant matrix and target metabolites. Operating at a moderately elevated temperature improves solvent diffusion and cell wall permeability, leading to higher extraction yields and greater efficiency than cold maceration, without reaching temperatures that would compromise thermolabile compounds Figure 7 illustrates both the digestion and decoction (Section 3.3) processes. However, temperature must be carefully optimized as excessive heating can promote degradation of heat-sensitive phenolics, glycosides, and volatile terpenoids [52].

In addition to temperature, extraction efficiency can be influenced by solvent pH and endogenous enzymatic activity within plant tissues. Acidic conditions (pH 2–5) promote the extraction of alkaloids and anthocyanins through protonation-mediated solubility enhancement, alkaline conditions (pH 8–11) favor phenolic acids and saponins, and neutral pH is generally preferred for broad-spectrum recovery of flavonoids and terpenoids [53]. In a hybrid technique, thermostable endogenous enzymes such as cellulases, hemicellulases, and pectinases remain catalytically active at temperatures below ~50 °C, contributing to partial cell-wall degradation and facilitating release of glycosylated secondary metabolites [54]. As temperature increases beyond 60–70 °C, progressive enzyme denaturation terminates this catalytic contribution, providing a practical control point when preparing extracts for downstream biological assays.

The impact of thermal activation on extraction efficiency was demonstrated in aqueous digestion of *Cocos nucifera* husk, where stepwise temperature increases from ambient to 50 and 70 °C yielded progressively higher phenolic content [55]. Extraction at 70 °C in three 0.5 h cycles afforded the highest antioxidant capacity and antibacterial efficacy against *Streptococcus mutans*, while temperatures approaching solvent boiling point led to reduced yields, indicative of phenolic degradation. These results identify 70 °C as the optimal temperature for balancing enhanced mass transfer with compound stability.

**Figure 8:**
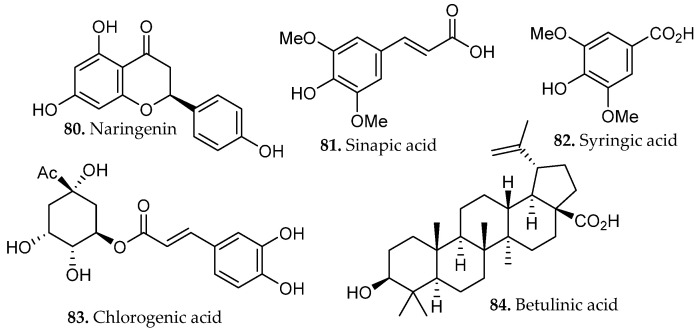
Natural products **80**–**84** extracted by digestion.

An optimized digestion-based extraction of *Sambucus nigra* (elderflower), a polyphenol rich plant matrix, was developed using a Box–Behnken response-surface design to evaluate solvent composition, extraction time, and solvent-to-solid ratio [56]. Solvent composition was identified as the dominant factor, with approximately 45% methanol yielding maximal recovery of polyphenolic constituents. Under these conditions, the extract contained elevated levels of rutin (**19**, Figure 1), isoquercetin (**22**, Figure 1), and chlorogenic acid (**83**, Figure 8). While extraction time remains an important consideration in extraction optimization broadly, its influence in this specific case was comparatively modest, with solvent polarity appearing to be the principal determinant of mass transfer efficiency. The optimized extracts demonstrated enhanced α-glucosidase and α-amylase inhibitory activity consistent with increased phenolic content, illustrating the value of statistically guided parameter optimization in digestion-based extraction.

A similar optimization strategy was applied to the digestion of *Dillenia indica* bark, a medicinal plant widely used in South and Southeast Asia [57]. Extraction parameters, including solvent composition, temperature, time, and solid-to-liquid ratio, were optimized using an integrated response surface methodology (RSM) and artificial neural network (ANN) framework. Statistical modeling identified optimal extraction conditions of 46 °C, 63% ethanol, and 0.8 h. Under these conditions, the extract showed enhanced recovery of phenolic metabolites such as esculetin (**37**, Figure 2), naringenin, sinapic acid, and syringic acid (**80**–**82**, Figure 8), along with increased levels of betulinic acid (**84**, Figure 8). Machine learning–augmented optimization frameworks are increasingly being integrated into natural product extraction workflows, particularly for complex plant matrices in which multiple interacting variables govern extraction efficiency and compound integrity. The close agreement between predicted and experimental extraction responses in the ANN model demonstrates the value of integrating data-driven optimization into digestion extraction workflows, providing a robust framework for producing compositionally reproducible extracts suitable for downstream pharmaceutical development.

Despite its kinetic advantages over maceration, digestion is unsuitable for thermolabile or volatile constituents and is best reserved for applications where operational simplicity and reproducibility are priorities. The mild, controlled heating conditions it employs represent a practical compromise between extraction efficiency and metabolite integrity, positioning digestion as a useful intermediate technique between passive soaking and more thermally intensive methods such as reflux or Soxhlet extraction.

### 3.3. Decoction

Decoction is a traditional aqueous extraction method that involves sustained heating of plant material in boiling or near-boiling water to recover water-soluble constituents (Figure 7B). Typically conducted at temperatures approaching 100 °C for durations ranging from minutes to several h, decoction enhances extraction of moderately to highly polar compounds, including phenolic acids, glycosides, flavonoid glycosides, tannins, alkaloid salts, and triterpenoid saponins. Elevated temperature increases extraction efficiency but may also induce chemical transformations such as ester hydrolysis or glycosidic cleavage, potentially altering metabolite profiles. Thermolabile compounds remain susceptible to degradation under prolonged heating. In addition, the aqueous environment promotes co-extraction of highly soluble matrix components, including carbohydrates and pigments, which can complicate downstream purification.

**Figure 9:**
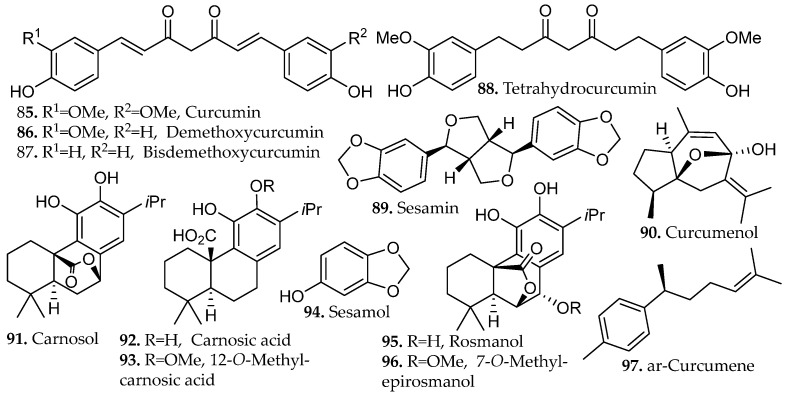
Natural products **85**–**97** extracted by decoction.

Decoction of *Curcuma longa* rhizomes was evaluated using a standardized protocol in which powdered material was boiled in water for 15 min, yielding an extract enriched in curcuminoids [58]. HPLC profiling confirmed curcumin (**85**, Figure 9) as the dominant constituent, accompanied by demethoxycurcumin, bisdemethoxycurcumin, tetrahydrocurcumin (**86**–**88**, Figure 9), curcumenol, and ar-curcumene (**90**, **97**, Figure 9), indicating that thermal treatment promoted both efficient solubilization of diarylheptanoids and the partial hydrogenation or endogenous reductive conversion of curcumin into reduced analogs. The extract exhibited strong antioxidant activity and robust anti-inflammatory activity consistent with the favorable binding affinity of these curcuminoids with target proteins involved in monkeypox virus replication and pathogenicity.

Decoction is highly suited for phenolic rich wood materials, as demonstrated in the chemical characterization of *Brunfelsia grandiflora* bark [59]. Seventy-nine phenolic compounds were identified in the decoction extract, including esculetin, scopoletin (**37**–**38**, Figure 2), sesamin, and sesamol (**89**, **94**, Figure 9). Roughly two-thirds of the detected phenolics were glycosides, which shows the ability of decoction to solubilize highly polar, sugar-conjugated phenolic structures. The rigid bark of *B. grandiflora* required sustained heating to disrupt matrix architecture and liberate tightly bound compounds, underscoring decoction as a suitable method for extracting phenolics from structurally dense tissues. The extract also exhibited strong antioxidant activity in DPPH and ABTS assays that were markedly superior to those reported for related *Brunfelsia* species and numerous *Asteraceae* plants. The strong radical scavenging capacity closely correlated with elevated phenolic content and indicates that the bark may serve as a potent source of antioxidant polyphenols with pharmacological potential.

A comprehensive evaluation for the decoction of three species rich in phenolic diterpenes, *Salvia officinalis*, *Salvia fruticosa*, and *Salvia rosmarinus* were recently reported [60]. A solvent-to-mass ratio of 2:150 (*w*/*v*) was reported for the extraction of *S. rosmarinus*. Carefully controlled decoctions significantly enhanced the release of abietane-type diterpenes such as carnosol, carnosic acid, 12-*O*-methylcarnosic acid (**91**–**93**, Figure 9), rosmanol, and 7-*O*-methyl-*epi*-rosmanol (**95**–**96**, Figure 9). In *S. fruticosa*, a five-minute decoction yielded approximately 50% more total diterpenes than infusion, with *S. officinalis* producing comparable results. *S. rosmarinus*, however, required an additional 15 min to reach maximum yields, likely due to the greater diffusional resistance imposed by its thicker, more resinous leaf architecture. These enhanced yields reflect two concurring factors, the increased aqueous solubility of oxygenated diterpenes at elevated temperatures, and the partial thermal conversion of carnosic acid to the more stable derivatives carnosol and rosmanol, transformations that proceed without significant degradation under short-term heating conditions.

Decoction is a thermally driven aqueous extraction strategy best suited for chemically robust plant materials whose target metabolites tolerate or benefit from near-boiling conditions. Sustained heating improves solvent penetration of dense plant matrices and accelerates the release of polar compounds, making the technique particularly effective for phenolic rich tissues that require prolonged thermal treatment for exhaustive extraction. However, decoction is unsuitable for volatile or thermolabile constituents, which are susceptible to heat-induced degradation. Overall, it represents a reliable and reproducible approach for the selective recovery of stable, water-soluble metabolites from complex plant matrices.

### 3.4. Percolation

**Figure 10:**
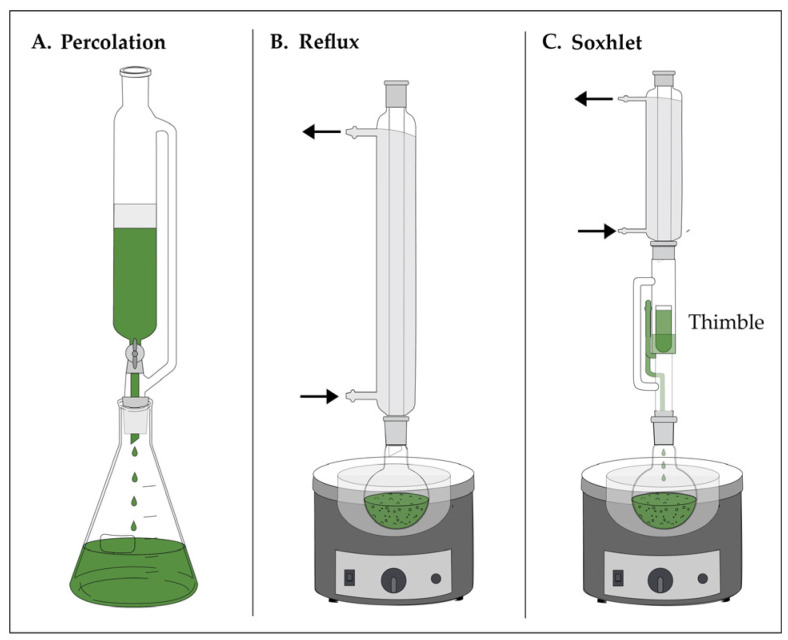
Standard experimental setups for conventional extraction techniques (**A**–**C**).

Percolation is a dynamic solid–liquid extraction in which solvent continuously passes through a bed of loosely ground plant material, sustaining a concentration gradient that enhances solute recovery. Following an initial pre-maceration step to ensure matrix hydration, solvent is passed through the column at a controlled flow rate to maintain continuous solvent renewal until the material is exhausted. Standard experimental setups for conventional extraction techniques including percolation, reflux (Section 3.5), and Soxhlet extraction (Section 3.6) are illustrated in Figure 10, with Figure 10A specifically depicting the percolation apparatus. Extraction efficiency depends on solvent polarity, particle size, percolation rate, bed porosity, and residence time [61]. Although continuous solvent renewal improves recovery relative to static extraction methods, percolation often requires substantial solvent volumes and precise flow rate regulation to ensure reproducibility. A solid-to-solvent ratio of 1:5 to 1:10 (g/mL) is commonly employed; however, this ratio is not universal and must be optimized based on the physicochemical properties of the target compounds, the nature of the plant matrix, and the solubility characteristics of the solvent system. Denser or more complex matrices may require higher solvent volumes to achieve adequate penetration and analyte release, while highly soluble constituents may be efficiently recovered at lower ratios. Empirical optimization of both solvent volume and flow rate is therefore essential to maximize extraction yield and ensure method reproducibility across different sample types.

The effectiveness of aqueous percolation for biological screening was demonstrated through extraction of antiparasitic plants from the Colombian Amazon, including *Abuta grandifolia*, *Ambelania duckei*, *Aspidosperma excelsum*, and *Curarea toxicofera* [62]. Dried and milled materials were first pre-moistened and macerated for 24 h before slow percolation (1 mL/min) until exhaustive extraction was achieved. A solid-to-solvent ratio of 1:10 (*w*/*v*) was employed for their percolation extraction protocol. Thin-layer chromatographic (TLC) profiling showed consistent recovery of flavonoids and steroids across all species, with alkaloids present in *A. grandifolia*, *A. excelsum*, and *C. toxicofera*, indicating effective solubilization of moderately polar metabolite classes under aqueous percolation. Extracts from *C. toxicofera* demonstrated the strongest concentration-dependent inhibition of *Trypanosoma cruzi* epimastigotes, whereas *A. duckei* displayed weaker activity and the remaining species were inactive at the tested range. These results highlight that percolation enables efficient metabolite recovery under mild conditions suitable for early-stage biological assessment.

**Figure 11:**
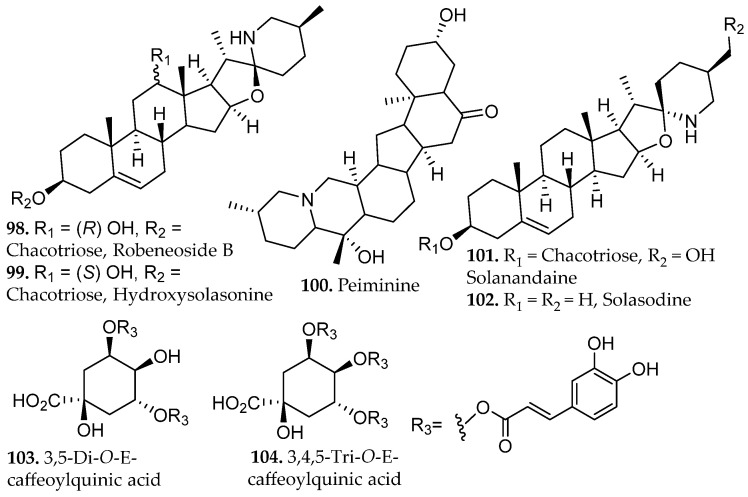
Natural products **98**–**104** extracted by percolation.

Ethanolic percolation of *Solanum lycocarpum* fruits demonstrated efficient recovery of the its major bioactive constituents, yielding greater extract mass than static maceration and providing a chemically enriched profile of steroidal glycoalkaloids such as robeneoside B, hydroxysolasonine, and solanandaine isomers (**98**–**99**, **101**, Figure 11) [63]. Additional analysis identified aglycone alkaloids peiminine and solasodine (**100**, **102**, Figure 11) and polyphenolic compounds such as 3,5-di-*O-E*-caffeoylquinic acid and 3,4,5-tri-*O-E*-caffeoylquinic acid (**103**–**104**, Figure 11). The extract displayed strong antioxidant, anti-inflammatory, and antinociceptive activities, consistent with the combined contributions of its abundant steroidal alkaloids and polyacylated phenolic acids that are well known to modulate oxidative, inflammatory, and nociceptive pathways. The continuous solvent flow in percolation likely prevents localized saturation inside the plant matrix, enhancing the release of bulky glycoalkaloids and limiting hydrolytic or oxidative processes that are more common during prolonged maceration or heated extraction.

Percolation’s principal strength lies in its ability to maintain a persistent solvent–matrix gradient, enabling continuous mass transfer and rapid depletion of accessible solutes under strictly ambient conditions. The uninterrupted flow of fresh solvent minimizes boundary-layer resistance, enhances desorption of high–molecular weight or amphiphilic metabolites, and reduces the residence time in solution that promotes hydrolytic or oxidative degradation.

Because extraction performance can be precisely modulated through particle size, solvent polarity, and flow rate, percolation provides reproducible kinetics and compositionally consistent outputs. These characteristics make it a technically robust method for applications requiring efficient solute transport, minimal thermal stress, and reliable chemical representativeness in both analytical and preparative natural product workflows.

### 3.5. Conventional Reflux Extraction

Reflux extraction is a classical solid–liquid technique in which solvent is continuously heated, vaporized, and condensed back into the extraction vessel, maintaining constant temperature and volume throughout (Figure 10B). The closed-loop design enhances extraction efficiency relative to static methods by sustaining solvent–matrix contact under controlled thermal conditions [64]. A wide range of solvents, including methanol, ethanol, acetone, chloroform, ethyl acetate, and *n*-hexane, may be employed depending on target metabolite polarity. Reflux extraction is effective for recovering heat-stable compounds but is unsuitable for volatile or thermolabile constituents.

**Figure 12:**
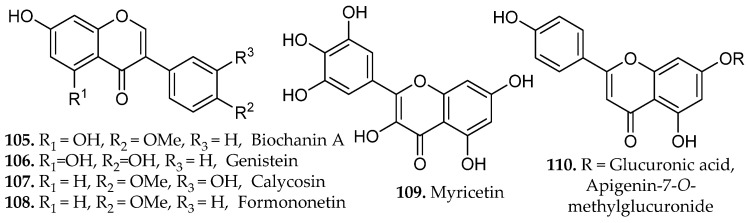
Natural products **105**–**110** extracted by conventional reflux extraction.

Reflux extraction of isoflavones from *Fabaceae* species showed that controlled temperature and solvent composition directly influenced both yield and bioactivity [64]. Factorial experiments varying methanol concentration, extraction time, and solid-to-liquid ratio revealed that prolonged heating near the solvent’s boiling point significantly increased total isoflavone content relative to shorter or lower-temperature extractions. HPLC quantification across twelve species, predominantly from the genus *Trifolium*, identified several isoflavone aglycones, including biochanin A, genistein, calycosin, and formononetin (**107**–**110**, Figure 12), as well as their corresponding glucosides, daidzin, ononin, genistin, and sissotrin (**24**–**27**, Figure 1).

A comparative study assessed maceration, decoction, reflux, and microwave-assisted extraction (MAE) for their effects on the phenolic composition and functional properties of *Gymnema inodorum* leaf extracts [65]. Ethanolic reflux extraction at 80 °C yielded the highest total phenolic content and total phenolic profile, reflecting the combined influence of elevated temperature and continuous solvent renewal on the efficient disruption of the leaf matrix and enhanced solubilization of semi-polar and polar phenolics. Chromatographic profiling revealed that reflux extraction yielded the highest relative abundance and diversity of phenolic constituents, including gallic acid (**58**, Figure 5), chlorogenic acid (**83**, Figure 8), sinapic acid (**81**, Figure 8), and myricetin (**109**, Figure 12).

In contrast, ethanolic maceration at room temperature yielded the highest flavonoid content, consistent with the heat sensitivity of this compound class. Enhanced phenolic recovery under reflux correlated with superior antioxidant capacity and enzyme inhibitory activity relative to other methods, demonstrating that reflux extraction offers a favorable balance of thermal energy and solvent polarity for liberating phenolic constituents from *G. inodorum* and supporting its utility as a chemically selective technique for phenolic-rich botanical matrices [65].

Hydroalcoholic reflux extraction of *Erigeron annuus* generated the most active antiviral fraction, outperforming maceration and UAE due to its superior recovery of mid-polar flavonoid glycosides and associated phenolic constituents [66]. Comparative extractions demonstrated that 50% ethanol under reflux produced the highest therapeutic index and yielded an ethyl acetate fraction enriched in flavonoids and polyphenols but lacking triterpenes and alkaloids.

Bioassay-guided fractionation identified apigenin-7-*O*-methylglucuronide (**110**, Figure 12) as the principal antiviral metabolite, isolated from the most active fraction. The compound demonstrated potent anti-RSV (Respiratory Syncytial Virus) activity with limited cytotoxicity against Human Epithelioma Type 2 (HEp-2, Hela cell cancer model positive for the presence of human papilloma virus), suggesting a favorable selectivity profile warranting further pharmacological investigation [66]. The results demonstrate that reflux extraction using alcohol-water mixtures is highly effective for extracting thermally stable plant compounds, especially flavonoids, which are difficult to obtain using milder extraction techniques.

Reflux extraction remains a thermally driven technique well suited for recovering mid-polar secondary metabolites whose solubility and diffusivity are enhanced under sustained boiling conditions. By matching solvent polarity and boiling point to the physicochemical profiles of target constituents, particularly phenolics, flavonoids, isoflavones, and triterpenoid saponins, reflux promotes continuous desorption and solvent penetration while maintaining structural fidelity for compounds stable to moderate heat. The closed, isothermal environment enables reproducible extraction kinetics, allowing solvent composition, duration, and temperature to be precisely tuned to maximize recovery of glycosylated aromatics and amphiphilic saponins while minimizing hydrolysis and oxidation.

When optimized, reflux extraction reliably yields chemically rich extracts from thermally stable plant matrices, making it one of the most dependable conventional extraction methods.

### 3.6. Soxhlet Extraction

Soxhlet extraction applies reflux principles within an automated, cyclical system that continuously renews solvent–solute equilibria, enabling exhaustive extraction of plant matrices with minimal manual intervention. The apparatus (Figure 10C) comprises a boiling flask, an extraction thimble containing finely milled plant material, and a condenser that returns solvent to the substrate in repeated siphoning cycles. Each cycle reestablishes contact between the matrix and fresh solvent, sustaining concentration gradients and promoting reproducible mass transfer of target metabolites.

Solvent selection governs both the thermal operating range and duration of heat exposure, as extraction proceeds at the solvent’s boiling point. While Soxhlet systems afford exhaustive, reproducible recovery of thermally stable constituents, prolonged boiling can degrade thermolabile metabolites, necessitating careful solvent selection and cycle optimization [67].

Methanolic Soxhlet extraction of *Syzygium aromaticum* buds delivered a higher recovery of chemically relevant metabolite classes compared with maceration and hydrodistillation, reflecting the combined influence of solvent polarity, sustained boiling-point temperatures, and continuous solvent renewal [67]. Methanol’s polarity and protic character favor solvation of the steroidal, triterpenoid, flavonoid, tannin, and phlobatannin classes identified by qualitative phytochemical screening, demonstrating strong solvent–metabolite complementarity with the dominant constituents of clove buds.

Concurrently, the continuous heating–condensation cycles intrinsic to Soxhlet extraction promote progressive disruption of plant cell matrices and sustained desorption of moderately polar secondary metabolites. The *n*-butanol subfraction exhibited consistent, dose-dependent antibacterial activity across all concentrations in *E. faecalis*, *S. aureus*, *K. pneumonia*, and *E. coli*. Although structural profiling was not pursued, the study’s qualitative phytochemical testing combined with extraction-method comparisons demonstrates that methanolic Soxhlet extraction efficiently mobilizes a broad spectrum of bioactive metabolite families from *S. aromaticum* and enhances antibacterial potency through solvent–structure complementarity and dynamic solvent cycling rather than through increased extract mass alone [67].

A comparative study of grape pomace from *Vitis labrusca* (Niagara Rosada) evaluated Soxhlet extraction, maceration, UAE, MAE, and pressurized liquid extraction (PLE) using ethanol as the solvent [68]. The study quantified extraction yield, total phenolic content, antioxidant activity (DPPH IC_50_), and/or other constituents by GC–MS to assess how extraction conditions influence both chemical composition and functional properties of the resulting extracts.

**Figure 13:**
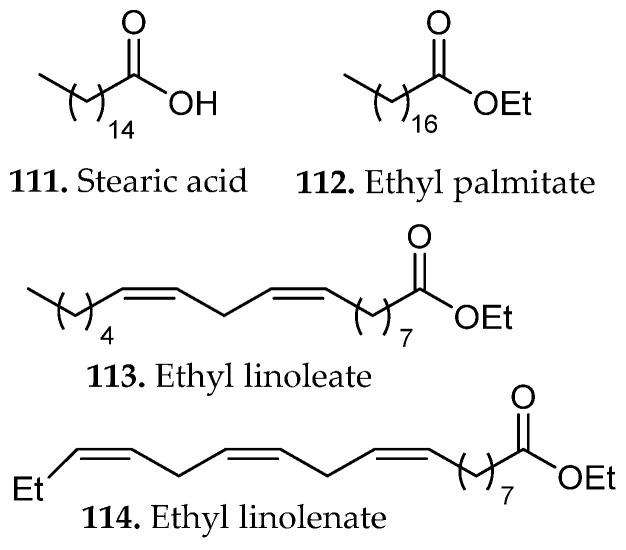
Soxhlet-extracted fatty acids (**111**–**114**).

Continuous solvent reflux and elevated temperature in Soxhlet extraction may facilitate the recovery of structurally distinct phenolic subclasses or lipophilic antioxidant constituents capable of stronger electron- or hydrogen-donating activity. GC–MS profiling showed that the extracts were dominated by fatty acids and fatty-acid ethyl esters, including palmitic acid, linoleic acid (**59**, **61**, Figure 5), ethyl linoleate, ethyl palmitate, stearic acid, and ethyl linolenate (**111**–**114**, Figure 13) [69]. These findings highlight that extraction methods selectively enrich different classes of compounds and should therefore be chosen according to the desired functional outcome of the extract.

Sequential Soxhlet extraction of *Morus alba* leaves with hexane, dichloromethane, ethyl acetate, and ethanol produced strongly polarity-dependent metabolite partitioning that governed both chemical composition and bioactivity [69]. Phytochemical screening and ^1^H-NMR data showed that the nonpolar hexane and dichloromethane stages were dominated by triterpenes and steroids, reflecting the high solubility of these hydrocarbon frameworks in low-polarity solvents.

All fractions except hexane contained phenolic compounds, flavonoids, and tannins, while alkaloids appeared only in the ethanol fraction generated under the gradient protocol. By removing nonpolar constituents prior to ethanol introduction, gradient Soxhlet extraction enhanced solvent efficiency, yielding the highest recovery of chlorogenic acid (**83**, Figure 8) and total phenolic content. The dichloromethane fraction, which was richest in flavonoids, exhibited the greatest cytotoxicity against HuCCA-1, MCF-7, and A549 cells, while the ethanol gradient extract demonstrated potent anti-migratory and anti-invasive activity against A549 cells with minimal effects on noncancerous HaCaT keratinocytes [69]. These outcomes illustrate how polarity-sequenced Soxhlet extraction separates terpenes from phenolic and flavonoid matrices, enabling targeted enrichment of distinct chemical classes and enhancing recovery of bioactive mid-polar phenolics.

Soxhlet extraction remains a methodological standard in natural product chemistry due to its solvent-reflux cycling mechanism, which sustains constant boiling-point temperatures and continuous solvent renewal. These conditions enhance diffusion, solubilization, and partitioning of lipophilic and mid-polar metabolites across successive extraction cycles. Strategic solvent selection based on physicochemical properties is critical, and sequential extraction using solvents of increasing polarity (e.g., *n*-hexane to ethyl acetate to methanol or ethanol) enables fractionation of distinct metabolite classes. The closed-loop reflux system provides reproducible extract profiles and near-exhaustive matrix depletion, reinforcing its continued use as a benchmark technique for comparison with emerging extraction technologies.

### 3.7. Steam Distillation

**Figure 14:**
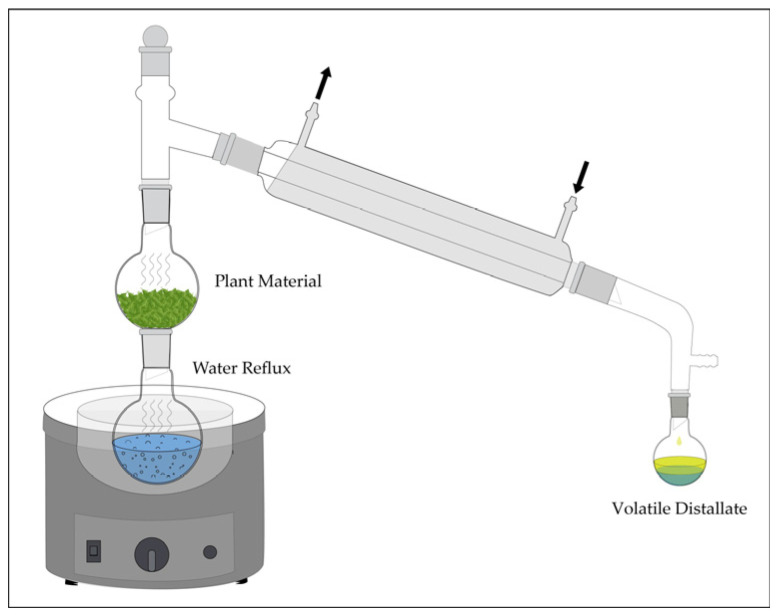
Steam distillation extraction diagram.

Steam distillation is a fundamental technique for isolating volatile natural products, especially essential oils, based on the principle that steam reduces the effective boiling points of immiscible volatile compounds through co-distillation. This allows extraction at temperatures below thermal degradation thresholds by lowering the partial vapor pressure of each component, enabling simultaneous vaporization with water while preserving heat-sensitive constituents. A typical steam distillation apparatus (Figure 14) comprises a round-bottom flask heated by a mantle, a distillation chamber packed with ground plant material, a condenser, and a receiver. Steam generated from the boiling water passes through the plant matrix, rupturing oil glands and volatilizing essential oil components.

The mixed vapors condense into a biphasic distillate comprising a lighter essential oil layer overlying an aqueous hydrosol phase; polar or water-soluble compounds may consequently be lost to the aqueous layer or apparatus surfaces. This classical method established the thermodynamic framework upon which modern solvent-free technologies, including microwave-assisted hydrodistillation and supercritical fluid extraction, have since been developed to improve selectivity, energy efficiency, and thermal stability [70].

**Figure 15:**
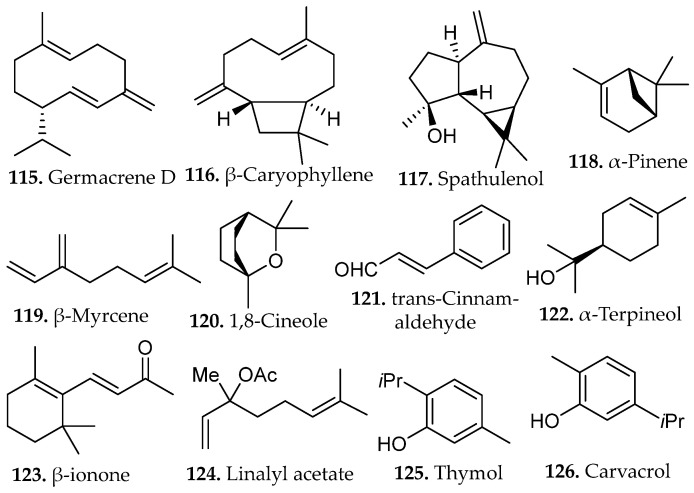
Natural products **115**–**126** extracted by steam distallation.

A systematic optimization of steam distillation parameters for *Kaunia longipetiolata*, an Andean species rich in sesquiterpenes, identified particle size reduction and distillation duration as key determinants of extraction efficiency [70]. Mechanical grinding enhanced steam penetration and volatile mass transfer, while a three-hours distillation period provided optimal metabolite recovery and chemical integrity. The resulting essential oil was dominated by germacrene D, β-caryophyllene, and spathulenol (**115**–**117**, Figure 15).

Extended distillation promoted mild oxidative transformation, generating caryophyllene oxide and spathulenol derivatives associated with increased antimicrobial activity against *Staphylococcus aureus* and *Candida albicans* [70]. These results demonstrate that distillation time influences both compositional profile and biological activity of sesquiterpene-rich extracts.

Steam distillation of *Acroptilon repens* further emphasized the necessity of process optimization [71]. One hour of extraction under constant vapor flux achieved maximal recovery of the nonpolar monoterpenes α-pinene, β-myrcene, and 1,8-cineole (**118**–**120**, Figure 15), all associated with insecticidal activity. Insufficient distillation time resulted in incomplete volatilization, while excessive duration promoted hydrolytic degradation and resinification, both detrimental to oil quality. The optimized extract demonstrated pronounced insecticidal activity against the greenhouse whitefly *Trialeurodes vaporariorum*, producing mortality rates more than fourfold greater than those observed under suboptimal extraction conditions. The chemical stability of these hydrocarbon monoterpenes under steam exposure is attributable to the absence of oxidatively labile carbonyl functionalities, which preserves both compositional integrity and biological potency throughout the distillation process.

Parallel optimization studies on volatile compounds extracted from cranberry processing residues demonstrated analogous trends [72]. Distillation at 100 °C for approximately one-hr yielded maximum essential oil recovery while preserving thermally sensitive aromatic components. The resultant oil was characterized by high concentrations of *trans*-cinnamaldehyde, α-terpineol, and β-ionone (**121**–**123**, Figure 15). Shortened distillation periods were insufficient to volatilize higher-boiling terpenoid alcohols, while prolonged thermal exposure promoted cinnamaldehyde polymerization, resulting in a concomitant loss of aromatic character. Extracts obtained under optimized conditions displayed significant antibacterial and antifungal activity against *Escherichia coli*, *Staphylococcus aureus*, and *Candida albicans*.

The observed bioactivity was attributed to the α,β-unsaturated carbonyl moiety of cinnamaldehyde, which facilitates nucleophilic addition with amino acid residues in microbial enzymes, resulting in enzymatic inhibition and cellular dysfunction [72]. These results underscore the requirement for precise thermal control to prevent degradation of electrophilic functional groups essential for antimicrobial activity.

Essential oils derived from *Thymus vulgaris*, *Salvia blancoana*, and *Lavandula intermedia* via steam distillation under systematically varied moisture content and extraction time revealed optimal performance at 1.5 h [73]. This protocol produced chemically balanced profiles enriched in linalyl acetate, thymol, carvacrol and β-caryophyllene (**124**–**126** and **116**, Figure 15). Prolonged distillation promoted oxidative conversion of terpenoid alcohols with measurable reduction in antioxidant capacity.

Optimally prepared oils exhibited potent antiparasitic activity against *Leishmania infantum* and *Phytomonas davidi*, primarily mediated by phenolic monoterpenes including thymol and carvacrol. It was suggested that these compounds exert cytotoxic effects through membrane disruption via interchelation into lipid bilayers, increasing permeability and initiating osmotic lysis [73]. The data confirms that extractions require rigorous control of hydration state and thermal exposure for maintaining the structural and biological properties of oxygenated terpenes.

When systematically optimized for temperature, vapor flux, and duration, steam distillation efficiently extracts and preserves volatile natural products susceptible to thermal degradation under conventional heating. The technique balances extraction yield with chemical stability by exploiting co-distillation thermodynamics that reduce vaporization temperatures while providing sufficient energy for volatile component release.

Properly executed steam distillation yields essential oils with well-defined chemical compositions, high concentrations of bioactive terpenes and aromatic aldehydes, and favorable characteristics for antimicrobial, insecticidal, and antiparasitic evaluation in early-stage pharmaceutical development. While conventional extraction techniques have demonstrated reliable efficacy, emerging modern technologies continue to address and overcome their inherent methodological limitations.

## 4. Modern Approaches

Emerging extraction technologies represent a paradigm shift toward environmentally sustainable methodologies, encompassing techniques such as pressurized liquid extraction (PLE), supercritical fluid extraction (SFE), ultrasound-assisted extraction (UAE), microwave-assisted extraction (MAE), pulsed electric field extraction (PEF), and enzyme-assisted extraction (EAE). These approaches demonstrate superior performance relative to conventional methods, including reduced solvent volumes, accelerated processing times, decreased energy consumption, and enhanced extraction yields.

Their classification as green technologies stems from adherence to sustainability principles through minimized hazardous waste generation and reduced environmental footprint while preserving or improving extraction efficiency. However, widespread implementation faces barriers related to specialized instrumentation requirements and substantial capital investment, particularly limiting accessibility in resource-limited research environments.

### 4.1. Pressurized Liquid Extraction

Pressurized liquid extraction (PLE) is a high-efficiency solid–liquid technique that applies elevated temperature (50–200 °C) and moderate pressure (10–20 MPa) to maintain solvents in the liquid state above their atmospheric boiling points, thereby enhancing solvation and accelerating mass transfer relative to maceration or reflux [74]. Under these pressurized conditions, reduced solvent viscosity and increased diffusivity enable rapid matrix penetration and efficient recovery of secondary metabolites that are often incompletely extracted under conventional atmospheric protocols.

**Figure 16:**
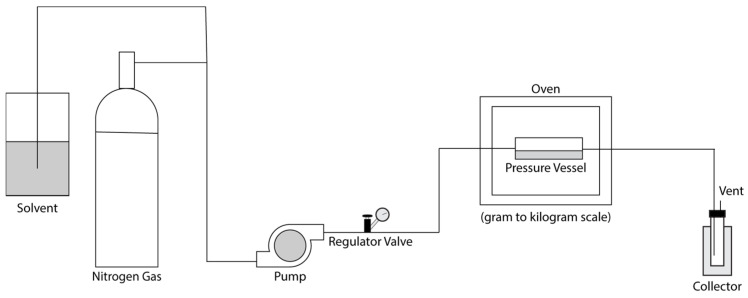
Schematic of a pressurized liquid extraction (PLE) system showing plant material placement within the pressure vessel (gram-to-kilogram scale).

A typical PLE system (Figure 16) consists of a solvent reservoir, high-pressure pump, preheater, stainless steel extraction cell, and collection vessel arranged in an automated sequential configuration. Fresh solvent is delivered by the pump into the heated extraction cell, where target analytes are solubilized during static or dynamic extraction cycles, and the depressurized effluent is subsequently collected downstream.

Systematic adjustment of temperature, pressure, and solvent composition enables selective enrichment of structurally distinct metabolite classes within a single extraction platform. Subcritical water and tailored co-solvent systems further expand accessible polarity ranges, reducing the need for multiple sequential extractions [74]. Compared to conventional techniques, PLE decreases extraction time and solvent consumption while offering reproducible, parameter-defined conditions that support standardization, metabolomic profiling, and bioactivity-guided isolation. These features have positioned PLE as a versatile and scalable alternative to Soxhlet and maceration in contemporary natural product workflows.

**Figure 17:**
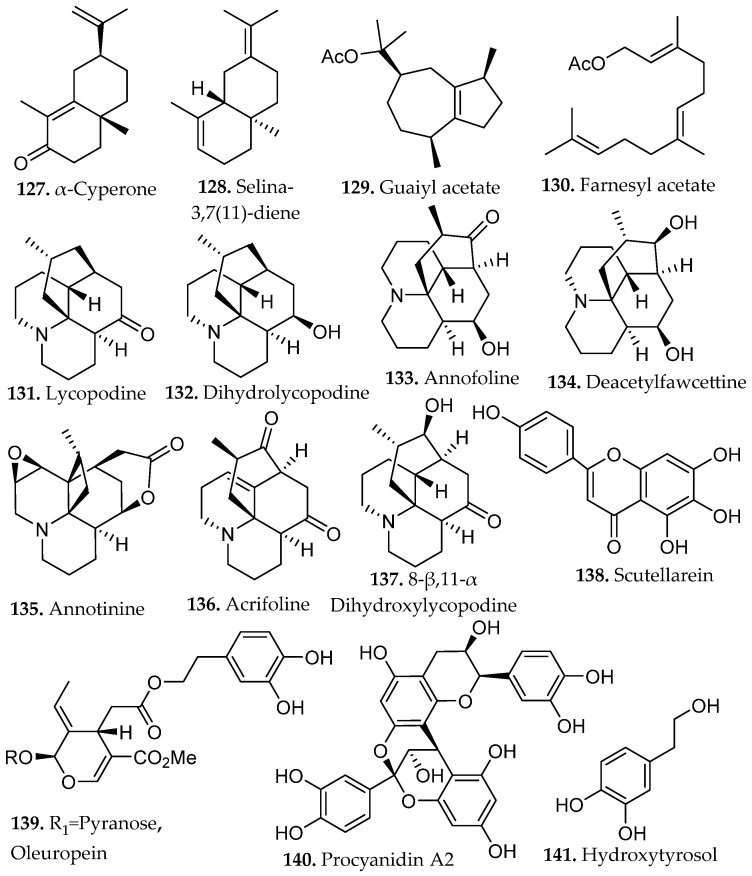
Natural products **127**–**141** extracted by pressurized liquid extraction (PLE).

PLE provided a chemically rational and tunable platform for isolating neuroactive metabolites from *Ferula persica* var. *latisecta*, with solvent polarity, temperature, and matrix composition jointly dictating solvation behavior and metabolite partitioning [75]. Initial screening with water, ethanol, ethyl acetate, and cyclopentyl methyl ether (CPME) showed that although water produced the highest mass yield from the aerial parts and roots, the most bioactive fractions consistently arose from the ethyl acetate and CPME fractions. Response-surface optimization identified an EtOAc:CPME mixture (79:21 *v*/*v*) at 180 °C as optimal for aerial tissues, maximizing extraction yield, total flavonoid content, and BChE inhibition. Optimal extraction of root tissues was achieved using 100% CPME at 180 °C, which afforded the highest total phenolic content and superior inhibitory activity against acetylcholinesterase and lipoxygenase, as well as enhanced reactive oxygen species (ROS) scavenging capacity. Untargeted LC/GC-MS profiling confirmed enrichment of sesquiterpenoids, monoterpenoid acetates, lignans, and hydroxycinnamates, with metabolites such as kaempferol (**32**, Figure 1), α-cyperone, selina-3,7(11)-diene, guaiyl acetate, and farnesyl acetate (**127**–**130**, Figure 17) correlating strongly with ROS scavenging and acetylcholinesterase inhibition. In contrast, supercritical fluid extraction (SFE) performed at 300 bar and 40 °C for 2 h yielded less than 1% extract mass and was not pursued further, underscoring the matrix-dependent advantages of pressure-stabilized liquid systems for recovering moderately polar and architecturally complex neuroactive constituents under optimized conditions.

Solvent polarity directly influenced the selective recovery of major *Lycopodium* alkaloids from *Lycopodium clavatum* and *Lycopodium annotinum* under optimized PLE conditions [76]. The targeted alkaloids included lycopodine, dihydrolycopodine, annofoline, deacetylfawcettine, annotinine, acrifoline, and 8-β,11-α-dihydroxylycopodine (**131**–**137**, Figure 17). The study employed four five distinct solvents representing a range of polarities: methanol (polar protic), acidified methanol with 1% tartaric acid 1% methanolic tar-taric acid (polar protic), ethyl acetate (moderately polar), dichloromethane (moderately polar aprotic), and cyclohexane (nonpolar).

Analysis of the resulting crude extracts revealed pronounced solvent-dependent alkaloid selectivity patterns. Acidified methanol provided the highest cumulative recovery of the full alkaloid profile, whereas dichloromethane selectively enriched lycopodine in *L. clavatum* and annotinine and acrifoline in *L. annotinum*. Nonpolar cyclohexane failed to solubilize detectable quantities of any of the monitored alkaloids in either species. Relative to earlier maceration and Soxhlet protocols across both species, PLE reduced solvent consumption and extraction time while enabling deliberate tuning of alkaloid selectivity through controlled solvent polarity [76].

Accelerated solvent extraction (ASE), synonymous with PLE, demonstrated superior efficiency for isolating the flavonoid scutellarein (**138**, Figure 17) from the leaves of *Triumfetta rhomboidea* when compared against three extraction methods, including simple maceration, Soxhlet extraction, and UAE [77]. All methods utilized ethanol as the extraction solvent to ensure fair comparison. ASE produced the highest overall extraction yield in addition to the highest concentration of the target compound scutellarein within the extract. In contrast, both maceration and Soxhlet extraction methods resulted in significantly lower scutellarein recovery. Although UAE is known to improve mass transfer relative to conventional techniques, its lower operating temperature and shorter extraction time may have constrained recovery of matrix-associated phenolics. The superior performance of ASE may be attributed to the elevated temperature and pressure-stabilized solvent environment, which can enhance matrix disruption and solubilization of mid-polar constituents.

PLE has recently demonstrated superior performance over UAE for the valorization of olive leaf agro-industrial waste as a source of bioactive polyphenols [78]. Under comparable green solvent conditions (15% ethanol or glycerol, 50–70 °C), PLE produced higher overall polyphenol recovery and antioxidant activity, with glycerol-modified systems at 70 °C providing the strongest performance. Biological evaluation further revealed significant α-glucosidase inhibition with minimal α-amylase inhibition, supporting the selective antihyperglycemic potential of the extracts. Chromatographic profiling showed that oleuropein (**139**, Figure 17) remained the dominant metabolite across all conditions, while PLE preferentially enhanced recovery of structurally complex flavonoids including quercetin (**20**, Figure 1), as well as procyanidin A2 (**140**, Figure 17) and resveratrol (**39**, Figure 2).

In contrast, UAE combined with glycerol more effectively enriched hydroxytyrosol (**141**, Figure 17), with additional recovery of gallic acid (**58**, Figure 5) and rutin (**66**, Figure 1) observed in polar solvent systems at elevated temperature [78]. The enhanced performance of PLE was attributed to the combined effects of elevated pressure and temperature, which improve solvent penetration, mass transfer, and solvation range relative to atmospheric ultrasound conditions, positioning PLE as an efficient and scalable strategy for industrial recovery of functional polyphenols from plant by-products.

PLE represents a controlled, thermodynamically intensified extension of conventional solvent extraction, using pressure-stabilized high-temperature liquids to expand solvation capacity beyond what is achievable at atmospheric conditions. Across diverse plant materials, the method consistently demonstrates that reducing solvent viscosity and increasing diffusivity improves access to mid-polar and hydrophobic metabolites embedded within lignocellulosic or resinous matrices, while tunable solvent polarity enables selective enrichment of distinct secondary metabolites. By matching solvent composition and operating temperature to the functional groups and ionization behavior of the target metabolites, PLE provides rapid isolation of natural products with markedly lower solvent consumption.

### 4.2. Supercritical Fluid Extraction

Supercritical fluid extraction (SFE) is an advanced, environmentally sustainable technique that exploits the unique properties of fluids above their critical temperature and pressure to isolate bioactive compounds from plant matrices. Carbon dioxide (CO_2_) is the preferred solvent due to its low toxicity, non-flammability, chemical inertness, and accessible critical parameters. Under supercritical conditions, CO_2_ provides low-viscosity, high-diffusivity transport that supports rapid extraction and good matrix penetration while limiting thermal degradation of target compounds [79].

Additional advantages include simplified downstream processing through facile CO_2_ removal upon depressurization and reduced oxidative risk. However, SFE requires specialized high-pressure instrumentation, which may limit accessibility. Because supercritical CO_2_ is inherently nonpolar, it is most effective for nonpolar to moderately polar metabolites. Recovery of highly polar natural products typically necessitates the addition of co-solvents such as ethanol or methanol. Incorporation of modifiers expands solvation capacity but introduces additional optimization variables, including co-solvent concentration and equilibration parameters, thereby increasing process-development complexity [80].

**Figure 18:**
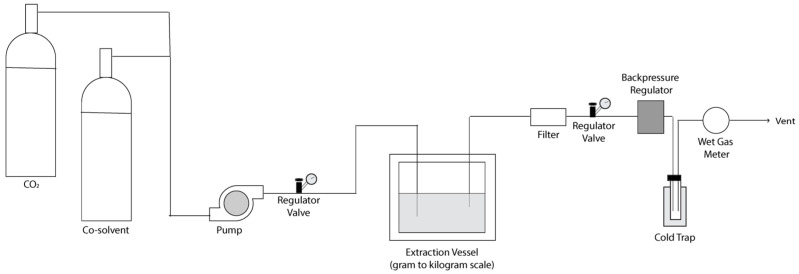
Schematic diagram of supercritical fluid extraction (SFE).

A typical SFE system (Figure 18) consists of a CO_2_ source, high-pressure pump, and pressure regulator that compress CO_2_ beyond its critical point. The pressurized CO_2_ is heated and pumped into a stainless-steel extraction vessel containing powdered plant material held by porous filters. As supercritical CO_2_ flows through the plant matrix, it dissolves target compounds. The extract-rich stream then passes through a backpressure regulator into collection vessels, where depressurization causes dissolved compounds to precipitate as CO_2_ returns to the gas phase. A cold trap captures the concentrated extracts while the gaseous CO_2_ is recondensed and recycled, creating an environmentally sustainable closed-loop system. Modern SFE systems also include co-solvent reservoirs [79].

**Figure 19:**
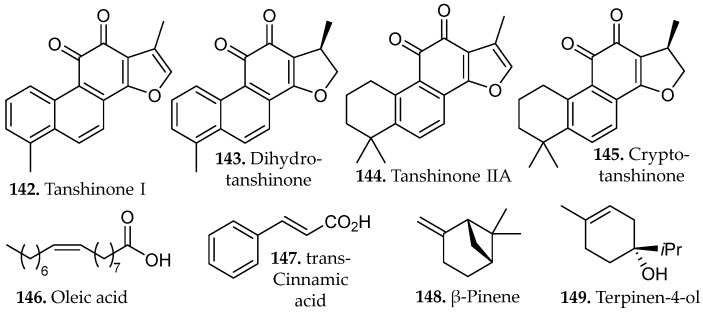
Natural products **142**–**149** extracted by supercritical fluid extraction (SFE).

Supercritical CO_2_ extraction employing peanut oil as a co-solvent significantly improved the recovery of tanshinone compounds from *Salvia miltiorrhiza* (Danshen) roots [81]. Using a Box–Behnken experimental design for systematic optimization, it was found that increasing peanut oil concentration up to approximately 50% consistently improved the solubility and extraction yield of all four target tanshinones, including tanshinone I, dihydrotanshinone, tanshinone IIA, and cryptotanshinone (compounds **142**–**145**, Figure 19), with tanshinone I and IIA showing the greatest enhancement. Under optimized conditions, this modified SFE approach achieved tanshinone concentrations exceeding those obtained using conventional extraction methods, including methanolic and ethanolic Soxhlet extraction, as well as ultrasound-assisted methanolic extraction.

Mechanistically, natural oil modifiers such as peanut oil broaden the effective polarity range of supercritical CO_2_ systems and improve mass transfer through resinous plant matrices. Their amphiphilic fatty acids, including oleic (C18:1) and linoleic acid (C18:2), enhance solubilization of moderately polar aromatic diterpenoids without reliance on volatile organic modifiers. SFE without modifier produced sharply lower recoveries for all compounds, and non-pressurized methods were limited by insufficient solubilization of the hydrophobic tanshinone core and longer diffusion paths [81]. The solvent-dependent enrichment profiles demonstrate that peanut oil modified SFE uniquely accommodates the mid-to-low-polarity abietane diterpenoids, delivering superior extraction efficiency, sharper selectivity, and markedly reduced processing time relative to conventional techniques.

Supercritical CO_2_ extraction of *Moringa oleifera* with tailored co-solvent systems illustrated how solvent polarity and phase behavior govern recovery of structurally distinct phenolics [82]. UHPLC-QToF (Ultra High-Performance Liquid Chromatography- Quadrupole Time of Flight) analysis identified apigenin, quercetin, and kaempferol conjugates, including two apigenin glucoside isomers, quercetin-3-*O*-glucoside (**21**, Figure 1), quercetin malonyl glucoside, quercetin acetyl glucoside, kaempferol-3-*O*-glucoside (**34**, Figure 1), and kaempferol malonyl glucoside. Neither pure supercritical CO_2_ nor CO_2_ modified with ethanol could extract detectable quantities of these flavonoid compounds. The addition of water fundamentally altered the system by creating a biphasic CO_2_–water mixture that substantially increased the polarity of the extraction medium, enabling recovery of the target compounds. Optimal performance was achieved with a 1:1 CO_2_:water ratio, which yielded the highest total flavonoid recovery. Further optimization indicated that reduced pressure combined with elevated temperature favored extraction of quercetin and kaempferol glucosides, with quercetin-3-*O*-glucoside and its malonyl ester remaining dominant across conditions. Time-course experiments showed that nearly the entire extractable flavonoid pool was recovered within two hours, demonstrating that water-modified supercritical CO_2_ can efficiently isolate glycosylated flavonols while minimizing reliance on organic solvents.

Supercritical CO_2_ extraction modified with ethanol demonstrated that operating pressure influenced the chemical composition of extracts obtained from *Glycine max* (soybean) fermented solids [83]. When comparing extraction methods, both Soxhlet extraction and ethanol-modified SFE (scCO_2_ + EtOH) yielded similar fatty acid profiles, with extracts predominantly containing linoleic acid (**63**, Figure 5) and oleic acid (**146**, Figure 19). This consistent pattern reflects the high solubility of long-chain unsaturated fatty acids in hot organic solvents under both extraction conditions. However, quantitative analysis revealed that the supercritical CO_2_–ethanol extracts consistently contained higher total phenolic content than Soxhlet extracts, particularly at elevated pressure and temperature. The resulting extracts were significantly enriched in phenolic compounds and aglycone isoflavones, including gallic acid (**58**, Figure 5), genistein (**106**, Figure 12), *trans*-cinnamic acid (**147**, Figure 19), and daidzein (**23**, Figure 1). This enhanced recovery is consistent with the physicochemical properties of the supercritical system: increasing pressure elevated CO_2_ density and solvent strength while maintaining low viscosity and high diffusivity, thereby improving mass transfer efficiency and promoting more effective solvation and desorption of moderately polar phenolic acids and aglycone isoflavones from the fermented matrix.

Supercritical CO_2_ extraction of *Zanthoxylum myriacanthum* produced a volatile profile enriched in nonpolar essential-oil constituents relative to hydro-distillation [84]. Under optimized conditions, supercritical CO_2_ yielded a higher mass of essential oil than hydro-distillation and produced an extract dominated by monoterpene hydrocarbons, with β-pinene (**148**, Figure 19) as the principal constituent alongside substantial *d*-limonene (**2**, Figure 1), α-pinene, β-myrcene (**118**–**119**, Figure 15), and terpinen-4-ol. (**149**, Figure 19). The nonpolar nature of supercritical CO_2_, combined with its enhanced density under pressure, creates a highly selective extraction medium that preferentially concentrates nonpolar monoterpenes and other valuable terpenoid compounds. Despite the lower total phenolic content of the supercritical CO_2_ extract, it demonstrated superior antioxidant activity in both DPPH and ABTS radical scavenging assays. This enhanced antioxidant performance is attributable to the elevated concentrations of specific monoterpenes, particularly β-pinene, β-myrcene, and terpinen-4-ol. These compounds possess conjugated double bond systems and allylic structural motifs that are highly effective at stabilizing free radical intermediates through electron delocalization. These observations highlight that maximal extract yield does not necessarily correspond to superior functional performance, as bioactivity depends on compositional distribution rather than total mass recovered.

SFE represents an evolution of the solvent-free extraction principles first established by steam distillation, offering precise thermodynamic control over temperature and pressure parameters while maintaining environmentally sustainable operation. This advanced approach enables the selective recovery of natural products across a spectrum from nonpolar to moderately polar metabolites through careful manipulation of supercritical CO_2_ density and solvating properties. The incorporation of co-solvent systems significantly expands the polarity range accessible to SFE while preserving its environmental advantages. Ethanol-water mixtures function as polar modifiers by increasing the dielectric constant of the supercritical phase and introducing hydrogen-bonding capacity, thereby enabling extraction of phenolic compounds, glycosides, and other polar metabolites that are poorly soluble in pure CO_2_. Natural oil modifiers, such as peanut oil, provide an alternative approach that can further broaden solvation behavior of target metabolites through enhanced interactions with amphiphilic fatty acids such as oleic acid (C18:1) and linoleic acid (C18:2).

### 4.3. Pulsed Electric Field Extraction

**Figure 20:**
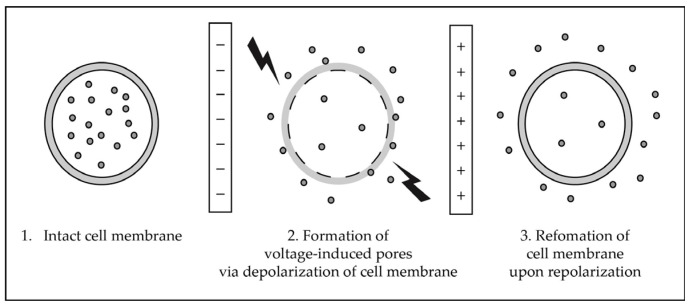
Diagram illustrating metabolite release via electroporation during PEF extraction.

Pulsed electric field (PEF) extraction is a non-thermal intensification technique that enhances mass transfer through electroporation-induced permeabilization of plant cell membranes, enabling the release of intracellular metabolites without extensive thermal input (Figure 20). The application of short, high-voltage pulses generates a transmembrane potential that exceeds the electrical endurance of the cell membrane, resulting in reversible or irreversible pore formation depending on field strength and pulse parameters [85]. This mechanism offers several advantages, including reduced extraction times, improved yields of thermolabile and oxidation-sensitive compounds, and lower solvent and energy consumption compared to conventional solvent-based techniques.

PEF treatment functions as an effective pretreatment strategy that enhances subsequent extraction processes, including solvent extraction, mechanical pressing, and diffusion-based methods. Its performance depends on careful optimization of electrical parameters tailored according to plant matrix properties and target compounds to achieve efficient membrane permeabilization without compromising compound integrity. The efficacy of PEF is influenced by the electrical conductivity and structural characteristics of the treated material. Moderately lignified tissues with adequate conductivity respond efficiently, whereas highly lignified or low-conductivity matrices may require higher field intensities or complementary pretreatments to facilitate effective membrane disruption. These pretreatment strategies can include moisture conditioning, ionic solution pretreatment, or hybrid approaches combining PEF with other cell disruption technologies [85].

A typical PEF system comprises a high-voltage pulse generator, an energy storage unit, and an extraction chamber equipped with two electrodes positioned to create a uniform electric field across the sample. The plant material, typically pre-moistened with a small volume of solvent to enhance electrical conductivity, is placed between the electrodes, where it is subjected to controlled electric pulses defined by field strength, pulse width, total number of pulses delivered, and pulse frequency. PEF systems may be operated in either batch or continuous-flow configurations. Batch systems are predominantly employed at laboratory and pilot scales, where smaller sample volumes and detailed parameter optimization studies are conducted. Continuous-flow configurations are favored for industrial applications, where higher throughput requirements and continuous processing capabilities are essential for commercial viability [85].

**Figure 21:**
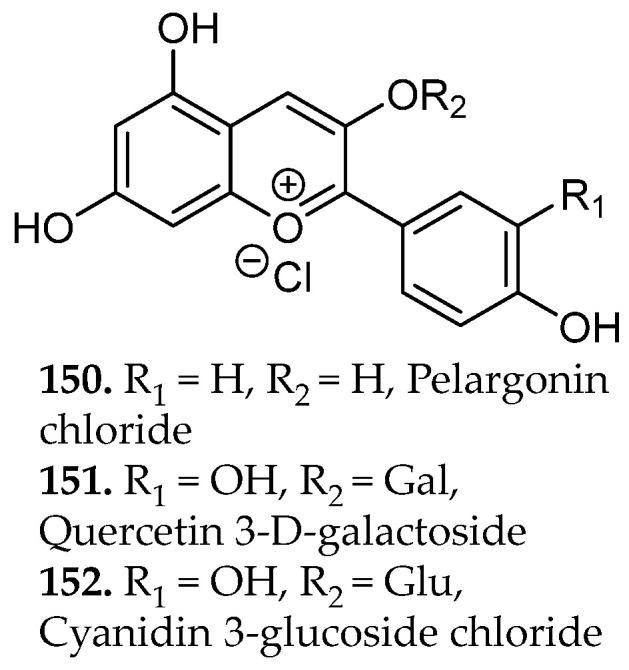
Natural products **150**–**152** isolated by PEF extraction.

An optimized PEF protocol developed for *Crataegus monogyna* leaf extraction demonstrated high recovery of polyphenols and strong antioxidant capacity under electrical and solvent parameters [86]. Response surface modeling confirmed that optimal extraction was achieved using 19% (*v*/*v*) aqueous ethanol (70 mL g^−1^) and PEF treatment at 1 kV cm^−1^ with 75 μs pulses for 10 min, producing extracts rich in pelargonin chloride, quercetin 3-*D*-galactoside, and cyanidin 3-glucoside chloride (**150**–**152**, Figure 21). These flavonoids are known for their anti-inflammatory and cardioprotective properties, and their selective enrichment illustrates the chemical control achievable through field tuning. This method produced consistent results across replicates, confirming both reproducibility and suitability for standardized phytochemical extraction in research and product development.

In the extraction of polyphenols from *Phyllanthus emblica*, PEF performance was shown to be strongly dependent on electric field strength, pulse number, treatment time, and solid loading [87]. Increasing electric field intensity enhanced polyphenol recovery up to an optimal threshold, beyond which extraction yields declined, indicating that excessive electrical stress likely promoted phenolic degradation or reduced effective mass transfer. A similar trend was observed for pulse number and treatment duration, where initial increases improved recovery but extended exposure resulted in diminishing returns, reflecting saturation effects and compound instability.

Solid loading also influenced extraction efficiency, with higher loadings decreasing performance due to reduced electrical homogeneity and conductivity within the treatment medium [87]. Under optimized conditions, this balance between effective permeabilization and compound preservation afforded higher polyphenol yields and antioxidant activity than both ultrasound-assisted and conventional aqueous extraction, underscoring the sensitivity of PEF outcomes to precise parameter control.

PEF extraction is a controllable intensification strategy in which electroporation-driven membrane disruption must be balanced against compound stability and matrix conductivity. Extraction efficiency depends on field strength, pulse architecture, and material-specific electrical and structural properties governing intra-system energy distribution. When optimized, PEF enhances metabolite diffusion and enables selective enrichment of bioactive compounds without the thermal burden of conventional techniques. Its compatibility with complementary extraction methods positions PEF as a precision-controlled platform for targeted phytochemical recovery rather than a simple strategy for maximizing yield.

### 4.4. Ultrasound-Assisted Extraction

**Figure 22:**
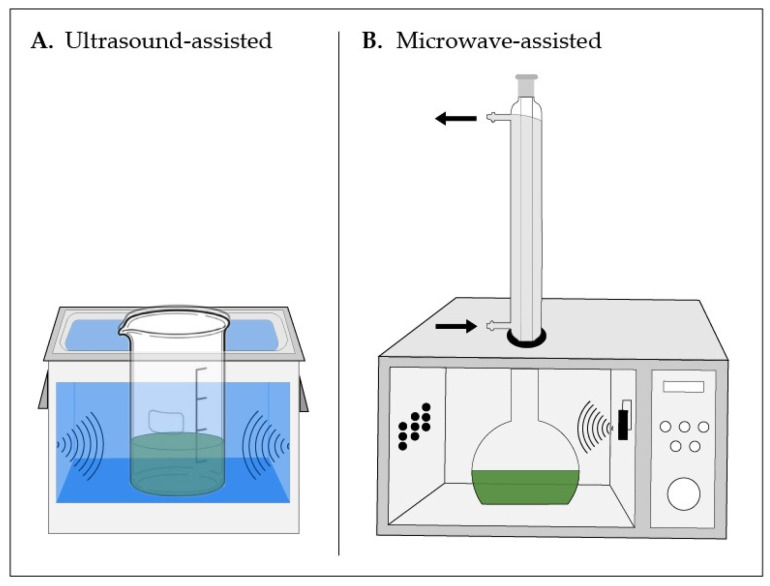
Representative experimental setups for modern extraction techniques including (**A**,**B**).

Ultrasound-assisted extraction (UAE) utilizes high-frequency acoustic energy to accelerate solvent–solid interactions through cavitation, or the formation and collapse of microbubbles in solvent. Representative experimental setups for modern extraction techniques UAE and microwave-assisted extraction (MAE, Section 4.5) are illustrated in Figure 22. Bubble implosion creates localized high temperature and pressure zones that disrupt cell walls and enhance solvent penetration, promoting rapid solute diffusion while maintaining low bulk temperatures that protect heat-sensitive compounds [88]. The combination of cavitation intensity, solvent polarity, extraction time, temperature, and solid-to-solvent ratio governs the overall extraction efficiency and reproducibility. UAE systems include either probe or bath configurations. Probe systems deliver concentrated acoustic energy directly into the extraction medium, suitable for resilient or highly lignified plant tissues, while UAE bath systems (Figure 22A) provide gentler, more uniform treatment for delicate matrices [88].

UAE enables substantially shorter extraction times and higher yields compared to conventional diffusion-driven methods while allowing operation at relatively low temperatures. Enhanced mass transfer often permits reduced solvent consumption and provides tunable selectivity through adjustment of ultrasound frequency and power [88].

UAE implementation requires consideration of equipment costs and maintenance, increased energy demand at larger scales, and achieving uniform ultrasound distribution in heterogeneous or viscous matrices. Careful optimization of ultrasonic intensity is also necessary to balance extraction efficiency with the stability of labile compounds.

In the extraction of phenolic compounds from aerial parts of *Fabiana punensis*, UAE outperformed MAE and matched conventional maceration in extraction efficiency while drastically reducing processing time [89]. UAE performance was primarily governed by the solid-to-solvent, followed by extraction time and ultrasound amplitude. Higher solvent volumes and longer sonication times enhanced phenolic and flavonoid recovery, whereas excessive amplitude reduced yields, likely due to localized heating and cavitation-induced degradation. Under optimized conditions (25% amplitude, 30 min, 1:20 g mL^−1^), UAE achieved total phenolic and flavonoid contents statistically comparable to maceration but in a fraction of the time.

**Figure 23:**
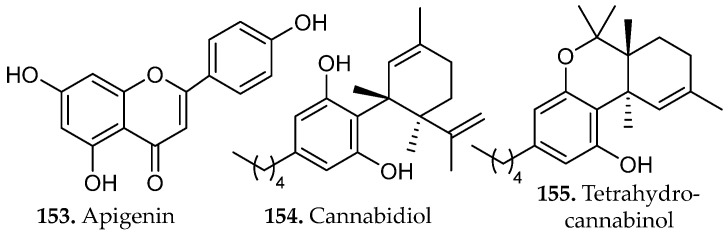
Natural products **153**–**155** isolated by ultrasound-assisted extraction (UAE).

Chromatographic analysis identified apigenin (**151**, Figure 23) as a major flavonoid constituent across extracts, and scanning electron microscopy revealed progressive cell-wall disruption and pore formation under ultrasound treatment, supporting cavitation-driven enhancement of mass transfer. UAE extracts also exhibited strong antioxidant and anti-inflammatory activity with low toxicity observed in multiple biological models, highlighting UAE as an efficient and scalable technique for recovering bioactive phenolics under mild conditions [89].

In the extraction of polysaccharides from *Epimedium brevicornum*, UAE was demonstrated to provide superior extraction efficiency relative to aqueous enzymatic extraction and microwave-assisted extraction when extraction parameters were carefully optimized [90]. UAE performance was strongly influenced by ultrasonic power, extraction time, temperature, and solid–liquid ratio, with each variable exhibiting a non-linear effect on polysaccharide yield. Increasing ultrasonic power enhanced recovery up to an optimal level (250 W), beyond which excessive cavitation reduced yields, likely due to polysaccharide degradation. Moderate extraction times and temperatures (60 min, 50 °C) maximized extraction efficiency without compromising structural integrity.

The ratio of solid plant material to liquid solvent was also critical, as sufficient solvent volume was required to ensure effective extraction while maintaining energy efficiency. When these conditions were optimized, UAE achieved the best polysaccharide recovery at almost 5% yield. The UAE extracts were also clearer and purer than those obtained using other extraction methods [90]. These findings establish UAE as an effective and scalable approach for polysaccharide extraction, provided ultrasound intensity and temperature are carefully controlled.

A representative example is the UAE-based extraction of phytochemicals from *Cannabis sativa* L. (hemp inflorescences), where extraction efficiency was strongly influenced by temperature, extraction time, and liquid-to-solid ratio, with compound-specific responses observed for cannabinoids and phenolics [91]. Using ethanol as the extraction solvent under controlled ultrasonic bath conditions (35 kHz), cannabidiol (CBD, **154**, Figure 23) recovery increased with temperature up to an optimal threshold (~20 °C), declining thereafter, indicative of sensitivity to cavitation- and temperature-induced degradation during prolonged sonication. In contrast, tetrahydrocannabinol (THC, **155**, Figure 23) extraction increased steadily with temperature, time, and solvent loading, reflecting compound-specific differences in mass-transfer behavior and thermal stability.

The liquid-to-solid ratio exerted a dominant influence on total phenolic and flavonoid recovery, with intermediate solvent volumes maximizing extraction efficiency, while excessive dilution provided limited benefit. Kinetic analysis further revealed divergent extraction mechanisms, with CBD following first-order kinetics, THC exhibiting diffusion-controlled behavior, and total phenolics displaying rapid initial release followed by diffusion limitation, underscoring the highly parameter-sensitive nature of UAE for complex phytochemical matrices [91].

Overall, UAE enhances the release and solubilization of bioactive molecules through controlled cavitation and solvent diffusion. The technique achieves high yields across phenolic, polysaccharide, and cannabinoid classes while preserving structural integrity. UAE therefore represents a scalable, chemically precise method that unites efficiency with selectivity, providing a valuable platform for both preparative and analytical extraction of natural products.

### 4.5. Microwave-Assisted Extraction

Microwave-assisted extraction (MAE) accelerates the release of intracellular metabolites by coupling electromagnetic energy directly into the solvent and plant matrix. Polar molecules within the solvent oscillate in the alternating electric field, generating heat through dipole rotation and ionic conduction. The rapid, uniform rise in temperature increases intracellular vapor pressure, rupturing membranes and enabling solute diffusion into the solvent. Unlike conventional heating, microwave irradiation produces volumetric heating throughout the sample, substantially reducing extraction time and solvent consumption while often enhancing yield relative to diffusion-controlled methods [92].

The shortened processing time of MAE limits prolonged thermal exposure of sensitive compounds, though excessive microwave power or irradiation duration may degrade highly thermolabile metabolites, and dense or heterogeneous matrices remain susceptible to non-uniform heating [92]. Scale-up requires consideration of microwave penetration depth and field distribution, and the specialized equipment involved introduces additional capital and operational considerations relative to conventional extraction techniques.

A standard MAE apparatus (Figure 22B) contains a magnetron generator that emits microwave radiation into a cavity holding the plant material immersed in solvent, which is operated in either open-vessel or closed-vessel configurations. Closed-vessel systems allow pressurized operation, enabling solvents to exceed their atmospheric boiling points and enhancing solubility and mass transfer, whereas open-vessel systems operate at ambient pressure and are preferred for heat-sensitive compounds. Solvent selection is a critical determinant of MAE efficiency, as microwave energy absorption depends strongly on dielectric properties. Polar solvents such as water and alcohols exhibit high dielectric loss and efficient heating, while non-polar solvents are largely inert to microwave radiation. Consequently, aqueous ethanol or methanol mixtures are most employed, providing effective microwave coupling alongside tunable polarity suitable for extracting a broad range of polar to moderately non-polar phytochemicals. Non-polar solvents may be used only in combination with polar co-solvents [92].

**Figure 24:**
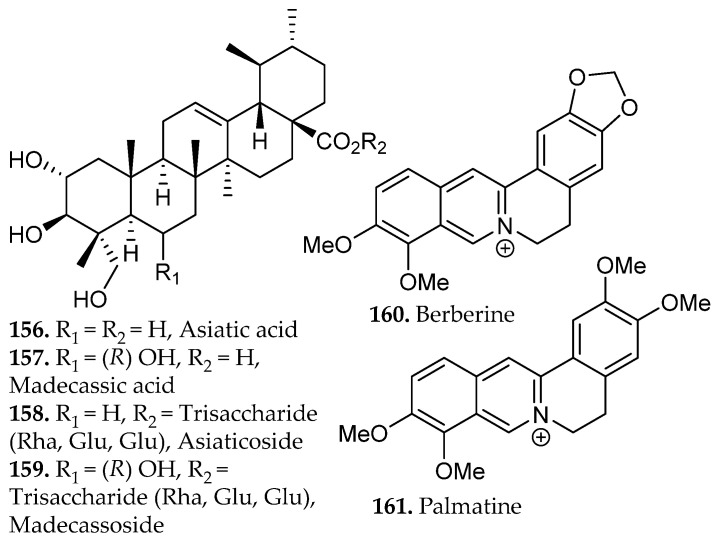
Natural products **156**–**161** extracted by microwave-assisted extraction (MAE).

Extraction efficiency in the MAE of *Centella asiatica* metabolites was primarily governed by solvent composition, solid-to-liquid ratio, and irradiation time under closed-vessel conditions [93]. MAE was performed using a pressurized microwave digestion system operating at effective powers up to ~600 W, where rapid dielectric heating promoted cell rupture and enhanced solubilization of triterpenes, including asiatic acid, madecassic acid, asiaticoside, and madecassoside (**156**–**159**, Figure 24).

Methanol-based systems were critical, with a 1:25 g mL^−1^ solid-to-liquid ratio and a 90% (*v*/*v*) methanol–water mixture yielding the highest total triterpene glycoside content. Extraction yields plateaued beyond 20 min, indicating rapid equilibrium attainment under microwave conditions. MAE achieved triterpene recoveries comparable to UAE and markedly higher than Soxhlet extraction, while requiring substantially shorter processing times and reduced solvent consumption. Although MAE did not surpass UAE in total yield, the results underscore the importance of solvent polarity and irradiation parameter optimization, as excessive energy input or suboptimal solvent selection can limit efficiency despite accelerated mass transfer [93].

The MAE of *Berberis jaeschkeana* and *Berberis asiatica* roots was optimized for the recovery of the primary alkaloid targets berberine and palmatine (**160**–**161**, Figure 24) [94]. Extraction efficiency was strongly governed by microwave power, solvent composition, solvent pH, and solvent-to-solid ratio, with pronounced interactive effects observed across response variables. Methanol proved the most effective solvent owing to its high dielectric loss, while acidic conditions (pH ≈ 2.0) were critical for maintaining alkaloid stability during microwave irradiation. Under optimized conditions (≈600 W, 2 min, 70 mL g^−1^, 100% methanol), berberine and palmatine yields were higher than yields with earlier reported conventional extraction approaches. MAE also enabled concurrent recovery of phenolic acids and flavonoids, including gallic acid (**58**, Figure 5), chlorogenic acid (**83**, Figure 8), vanillic acid (**52**, Figure 4), caffeic acid (**42**, Figure 2), and rutin (**19**, Figure 1), with antioxidant responses closely matching predicted values.

MAE is governed by the interplay of solvent properties, microwave power, and irradiation time. Rapid internal heating and pressure development accelerate mass transfer, offering a distinct kinetic advantage over conventional techniques, yet extraction efficiency remains sensitive to energy input and solvent composition, as excessive irradiation or mismatched solvent systems can promote degradation or reduce selectivity. When these parameters are aligned with target compound chemistry, MAE enables rapid equilibrium attainment and recoveries comparable to or exceeding those of conventional and ultrasound-assisted methods, with utility for thermally stable metabolites.

### 4.6. Enzyme-Assisted Extraction

Enzyme-assisted extraction (EAE) employs specific hydrolytic enzymes to degrade structural components of plant cell walls, facilitating release of metabolites entrapped within lignocellulosic matrices. Targeted enzymes act on cellulose, hemicellulose, pectin, starch, proteins, and lipid-associated structures, increasing matrix porosity and weakening intermolecular interactions such as hydrogen bonding and hydrophobic associations. In some systems, enzymatic modification can also enhance extractability by converting lipophilic compounds into more water-compatible forms through glycosylation [95]. This controlled biocatalytic disruption enables improved solvent penetration under mild conditions, reducing the need for elevated temperatures or harsh chemical treatments.

**Figure 25:**
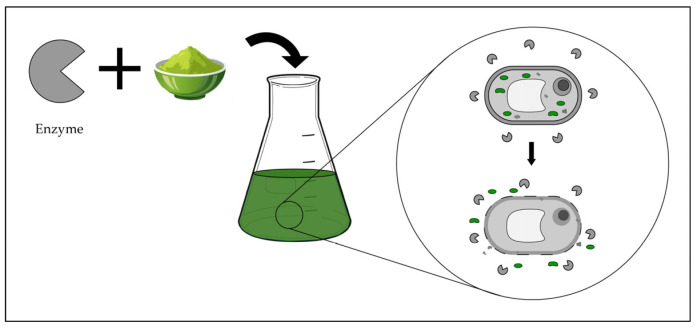
Schematic representation of enzyme-assisted extraction and its operational mechanism.

A typical EAE setup (Figure 25) consists of a temperature-controlled vessel in which finely milled plant material is suspended in an aqueous or buffered medium, most commonly within a mildly acidic pH range (4–6) to coincide with the isoelectric point of the enzyme. Enzymes such as cellulase, pectinase, hemicellulase, amylase, or multi-enzyme complexes (e.g., carbohydrases) are added at defined concentrations, and the mixture is gently agitated at moderate temperatures (30–55 °C) for a controlled incubation period [95].

EAE is particularly advantageous for recovering thermolabile or matrix-bound bioactive compounds and can improve extract clarity by degrading co-extracted macromolecules such as polysaccharides and proteins [95]. While EAE offers advantages such as improved yields, reduced thermal degradation, and the use of benign solvents (e.g., water), its limitations include enzyme cost, sensitivity to pH and temperature, and the need for careful parameter optimization. Consequently, EAE is most effectively implemented as a pretreatment strategy that enhances mass transfer and extraction efficiency when integrated with complementary physical or solvent-based extraction techniques [95]. Following enzymatic pretreatment, the system is often coupled with a downstream extraction step, such as conventional solid–liquid extraction, UAE, MAE, SFE, or cold pressing, to recover the solubilized metabolites.

EAE was applied to *Verbascum nigrum* L. to enhance recovery of phenolic and flavonoid constituents through targeted cell-wall disintegration [96]. A two-step protocol employed a lignocellulolytic enzyme cocktail (cellulase, pectinase, xylanase, and α-amylase) as a controlled pretreatment prior to hydroalcoholic extraction. Enzymatic pretreatment significantly improved extraction efficiency, achieving higher total phenolic and flavonoid yields within 2 h, whereas hydroalcoholic extraction alone failed to reach comparable recoveries even after 24 h. Design-of-experiments (DOE) analysis identified pH, temperature, enzyme concentration, and treatment time as compound-dependent variables: acidic pH and elevated temperature favored phenolic recovery, while slightly higher pH and extended treatment enhanced flavonoid release. Cellulase and pectinase emerged as the principal drivers of matrix disruption, though enzyme loading beyond optimal levels produced diminishing returns.

**Figure 26:**
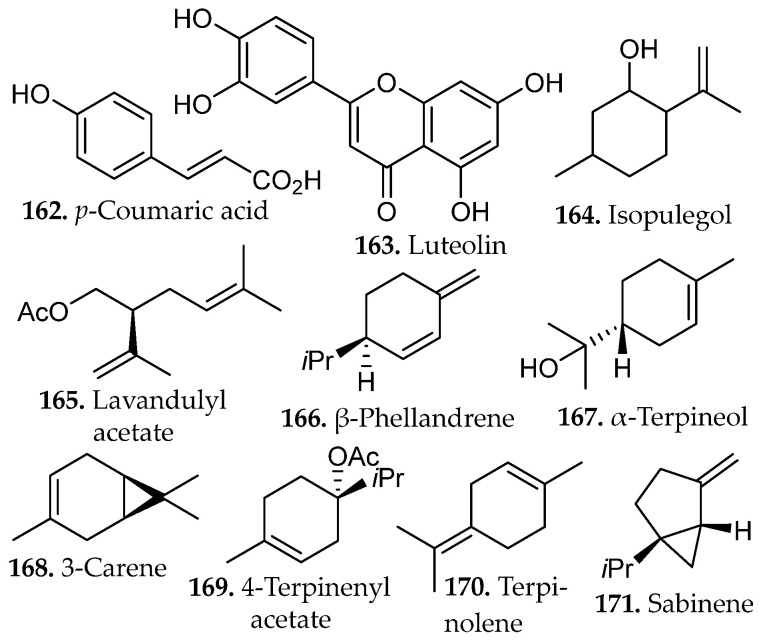
Natural products **162**–**171** extracted by enzyme-assisted extraction (EAE).

Chromatographic analysis confirmed substantially enhanced release of caffeic acid (**42**, Figure 2), *p*-coumaric acid, luteolin (**162**–**163**, Figure 26), and verbascoside (**66**, Figure 5). Notably, selected glycosylated phenylethanoids were partially diminished, reflecting enzyme-dependent compositional remodeling attributable to selective glycosidic cleavage, an outcome that underscores the precision with which enzymatic activity reshapes extract profiles [96]. Collectively, these findings affirm that EAE achieves enhanced solute liberation through controlled matrix disassembly, with practical performance governed by enzyme cost, incubation requirements, and the inherent susceptibility of individual compounds to hydrolytic transformation.

Leaves from *Cinnamomum longepaniculatum* were subjected to EAE as a pretreatment prior to hydrodistillation to evaluate how enzyme selection and treatment conditions influenced essential-oil yield, matrix disruption, and downstream bioactivity [97]. Under fixed screening conditions (enzyme treatment at 50 °C for 1 h followed by 2 h hydrodistillation), all enzyme pretreatments increased essential oil recovery relative to hydrodistillation alone. The β-glucanase/hemicellulase (1:1) combination produced the highest yield, consistent with enhanced deconstruction of structural polysaccharides within the cell wall matrix. Response surface methodology identified optimal parameters at pH 5, 1.12 h treatment time, and 48.5 °C, which maximized essential-oil yield and corresponded to conditions favorable for enzyme activity. Chemical profiling indicated that the essential oils remained rich in terpene compounds regardless of treatment, containing *dl*-isopulegol, β-phellandrene, α-terpineol, 3-carene, 4-terpinenyl acetate, lavandulyl acetate, terpinolene, and sabinene (**164**–**171**, Figure 26). However, shifts in relative chemical abundance were observed depending on which treatment was used. For example, *dl*-isopulegol remained predominant, whereas β-phellandrene and terpinyl acetate varied significantly with pretreatment, indicating that EAE parameters influenced not only yield but also compositional distribution of the recovered oil.

EAE functions as an extraction strategy in which selective enzymatic degradation of structural polysaccharides enhances matrix permeability and solute accessibility. Its effectiveness depends on precise control of pH, temperature, enzyme composition, and incubation time, as these variables govern both compound release and susceptibility to hydrolytic modification. While enzymatic pretreatment can substantially improve extraction efficiency and alter compositional distribution, performance remains bounded by economic considerations and compound-specific stability constraints. When carefully optimized and integrated with complementary techniques, EAE provides a controlled framework for enhancing phytochemical accessibility and tailoring extract composition within complex plant matrices.

## 5. Combined Techniques

Combined, or hybrid, extraction strategies have emerged as a logical extension of modern process intensification, pairing two or more complementary techniques to address limitations inherent to single-method workflows. Across recent studies, these approaches commonly exploit a mechanistic division of labor in which one technique disrupts matrix architecture or weakens solute–matrix interactions, while the second enhances solvation or mass transfer to recover target compounds more efficiently. Common examples include microwave–ultrasound hybrid extraction, microwave-assisted enzymatic extraction, and enzyme-assisted supercritical fluid extraction. In these systems, improved yields arise from increased solvent accessibility to intracellular domains, hydrolysis of structural polysaccharides or esterified linkages, and expansion of the effective extraction window available to the medium. These combinations can reduce extraction times and improve recovery relative to single techniques, particularly for mid-polar phenolics, alkaloids, and tightly bound terpenoids.

Various compelling illustrations of synergistic intensification are found in ultrasound- and microwave-based hybrid strategies, which enhance extraction efficiency through the coordinated application of thermal and mechanical forces. Rather than relying on a single mode of energy input, these approaches exploit the complementary mechanisms of rapid dielectric heating and cavitation-driven disruption to destabilize the plant matrix prior to or during solvent contact, facilitating more efficient solute mobilization.

Mechanistically, microwave irradiation generates rapid internal heating and pressure gradients within plant tissues, while ultrasound promotes cavitation and microstreaming that enhance solvent penetration and mass transfer. A comparable synergistic effect was demonstrated in microwave–ultrasound hybrid extraction of coffee pulp, where coordinated dielectric heating and cavitation-enhanced transport elevated procyanidin recovery beyond that achievable by MAE or UAE independently [98].

Alternatively, enzyme-assisted hybrid systems operate through selective biochemical modification of the lignocellulosic framework rather than purely physical disruption. In olive pomace, integration of microwave processing with enzymatic hydrolysis using tannase, cellulase, and pectinase increased phenolic extraction by promoting structural biotransformation of the matrix and improving enzyme access under intensified conditions [99].

Enzymatic depolymerization also reduces matrix rigidity and weakens barriers to solute release, thereby lowering the resistance to desorption during subsequent microwave or solvent extraction. Enzyme–ultrasound-assisted extraction in mangosteen peel, improved the co-recovery of phenolics and polysaccharides relative to ultrasound–microwave extraction, indicating that biochemical preconditioning can outperform purely physical disruption when target metabolites are tightly associated with structural carbohydrates [100]. In these systems, enzymatic treatment shifts the extraction-limiting step away from matrix integrity and toward solvent compatibility and mass-transfer kinetics, allowing intensified extraction techniques to operate more efficiently.

Optimal extraction is inherently target-specific, governed by metabolite polarity, thermal stability, molecular size, and matrix-binding state. Rational combination of complementary techniques aligns extraction kinetics with matrix chemistry, improving yield, selectivity, and efficiency establishing hybrid extraction as a cornerstone of modern natural products research.

## 6. Recent Advancements

Recent advancements in natural product research increasingly focus on two complementary domains: the development of environmentally responsible solvent systems and the integration of machine learning–driven optimization and discovery platforms. Green solvent innovations redefine extraction media by offering tunable solvation environments capable of enhancing selectivity, improving recovery of structurally elaborate metabolites, and reducing environmental burden relative to conventional organic solvents. In parallel, machine learning and artificial intelligence frameworks address downstream bottlenecks in dereplication, extraction optimization, and compound prioritization, enabling predictive modeling of extraction parameters and rapid annotation of metabolomic data. Together, these advancements shift natural product workflows from empirically guided solvent selection and labor-intensive isolation toward rationally designed, data-informed systems that improve chemical fidelity, sustainability, and efficiency in bioactive compound discovery.

### 6.1. Green Solvents

Green solvents, such as ionic liquids (ILs), deep-eutectic solvents (DES), natural deep-eutectic solvents (NADES), and bio-based solvents, not only replace hazardous organic solvents but actively enhance conventional extraction efficiency. Their tunable polarity and solvation potential improve solubility and selectivity for diverse phytochemicals, while water-modified systems reduce viscosity and accelerate mass transfer during maceration or Soxhlet extraction [101]. Low volatility and thermal stability protect thermolabile compounds across low- and high-temperature operations. Compatibility with standard laboratory equipment enables direct substitution without process redesign, providing immediate environmental and operational benefits. Thus, the integration of ionic liquids, DES, NADES, and bio-based solvents transforms traditional extraction processes into high-yield, low-impact, and chemically selective green alternatives.

#### 6.1.1. Ionic Liquids

**Figure 27:**
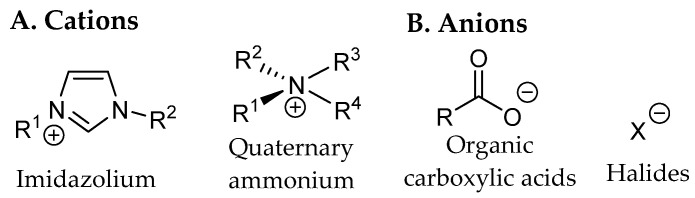
Examples of cations and anions used in the formation of ionic liquids.

Ionic liquids (ILs) are salts composed of bulky organic cations (e.g., quaternary ammonium, phosphonium, or sulfonium) paired with closed-shell anionic species (e.g., halides, phosphates, or borates) (Figure 27). These compounds exhibit several advantageous properties including high thermal stability, negligible vapor pressure, melting points below 100 °C at standard pressure, and tunable polarity through substituent modification. Furthermore, ILs can be recycled multiple times with minimal loss of performance, making them attractive alternatives to conventional organic solvents.

For extraction applications, imidazolium-based ILs have garnered substantial attention. Generally, increasing the anion size decreases viscosity, while elongating the alkyl chain promotes micelle formation through enhanced hydrophobic interactions, resulting in higher viscosity and improved solubility for hydrophobic analytes. Compared to traditional organic solvents, ILs demonstrate superior efficacy in extracting metabolites from plant cells, likely attributed to their ability to disrupt hydrogen bonding networks within the cellulose structure of plant cell walls [101]. This unique property positions ILs as powerful extraction media for plant-derived compounds.

The utility of ILs in UAE has been demonstrated through sequential treatment of [C_2_MIM]Cl followed by 1-decyl-3-methylimidazolium chloride [C_12_MIM]Cl to significantly enhance protein extraction yields from tobacco leaves compared to conventional extraction methods [102]. This sequential IL approach has proven to be broadly applicable across multiple plant matrices. When applied to golden tea, perilla, and mint leaves, the IL-based method achieved superior protein yields relative to traditional sodium dodecyl sulfate (SDS) and urea-based extraction strategies.

In a similar investigation, UAE performed with 1-dodecyl-3-methylimidazolium bromide ([C_12_MIM]Br) as the extraction solvent yielded both quantitative and qualitative improvements in lignan extraction compared to methanolic extraction or aqueous two-phase systems (ATPS) comprised of ethanol and ammonium sulfate [103]. The IL-based method demonstrated extraction times two- or three-fold shorter than UAE with methanol or ATPS, while simultaneously increasing lignan yields. Moreover, extracts obtained via the IL method exhibited enhanced radical scavenging activity, suggesting preservation of bioactive compounds during the extraction process.

Despite these promising applications, several critical challenges continue to impede the widespread adoption of ILs in biomolecule extraction. Three primary concerns have been identified: economic feasibility, environmental toxicity, and biodegradability. ILs remain relatively expensive compared to conventional organic solvents, which can limit their implementation in large-scale industrial processes. IL toxicity has been documented across diverse biological systems with varying severity [104]. Structure-activity relationship (SAR) studies indicate that toxicity is influenced by multiple structural features: (1) cationic head group identity, with aromatic cations (e.g., imidazolium, pyridinium) generally exhibiting higher toxicity than aliphatic counterparts; (2) alkyl chain length, where longer chains correlate with increased toxicity; and (3) anionic component, with polyfluorinated anions typically enhancing toxic effects.

Environmental persistence represents a significant limitation, as industrial-scale IL usage could result in aquatic contamination through wastewater discharge. Imidazolium-based ILs have demonstrated poor biodegradability in standard 5-day biochemical oxygen demand (BOD_5_) assays, suggesting potential for long-term environmental accumulation [104]. Recognition of these limitations has motivated the development of next-generation solvent systems designed to retain the beneficial extraction properties of ILs while mitigating their environmental and economic drawbacks. These advanced formulations are discussed in the following sections.

#### 6.1.2. Deep Eutectic Solvents and Natural Deep Eutectic Solvents

Deep eutectic solvents (DES) are binary or ternary mixtures that exhibit melting points significantly lower than their individual components. This phenomenon results from strong hydrogen bond donor (HBD) and hydrogen bond acceptor (HBA) interactions, creating dense molecular networks with unique physicochemical properties, including diverse solubility profiles, negligible volatility, and high thermal stability [105]. DES are classified into four types based on their constituents: Type I (organic salt + metal salt), Type II (organic salt + metal salt hydrate), Type III (organic salt + HBD), and Type IV (metal salt + HBD) representatives are highlighted in Table 1. For natural product extraction, Type III DES predominate, combining an ionic component with a hydrogen bond donor. This versatility enables fine-tuning of solvent polarity, selective metabolite interactions, cost-effectiveness, and reduced toxicity.

When both components are naturally sourced, these systems are termed natural deep eutectic solvents (NADES), such as choline chloride-urea formulations. Notably, many examples of DES extractions in literature use NADES but do not explicitly refer to the solvent as such. With the increased utilization of NADES in literature, understanding their mechanism in natural product extraction is essential.

The extractions of pectin from jack fruit wastes were compared between ultrasound-microwave assisted NADES extraction and the traditional ethanolic HCl extraction method [106]. ChCl based NADES prepared with HBDs tartaric acid, maleic acid, malonic acid, fructose, sucrose, and dextrose (1:1) were each evaluated. The NADES method increased pectin yield relative to the acidic ethanolic extraction. Enzymatic and supercritical CO_2_ extraction methods previously reported produced significantly lower pectin extraction yield when compared to the NADES method used in this study.

NADES composed of choline chloride and lactic acid were evaluated as biobased alternatives for anthocyanin extraction from Amazonian matrices including açaí, mangosteen pericarp, and purple yam [107]. While acidified hydroalcoholic solvents (70% methanol or ethanol with HCl) consistently achieved higher absolute anthocyanin yields, NADES systems demonstrated competitive recovery under optimized conditions when viscosity was reduced through controlled water addition and ultrasound assistance. Extraction efficiency depended strongly on NADES composition, water content, and ultrasound parameters, reflecting the sensitivity of anthocyanin solubility to hydrogen-bonding networks and solvent microstructure.

Importantly, despite lower yields for less polar or acylated anthocyanins, NADES-based extractions achieved substantially improved environmental performance, as quantified by a scale for the measurement of environmentally responsible consumers (Ecoscale) and Analytical GREEnness (AGREE) metrics, owing to reduced toxicity, elimination of mineral acids, and avoidance of volatile organic solvents [107]. These results highlight that NADES performance must be evaluated alongside sustainability metrics, where biobased solvent systems can offer meaningful advantages for food- and pharmaceutical-relevant extracts even when conventional solvents remain superior in raw extraction efficiency.

**Figure 28:**
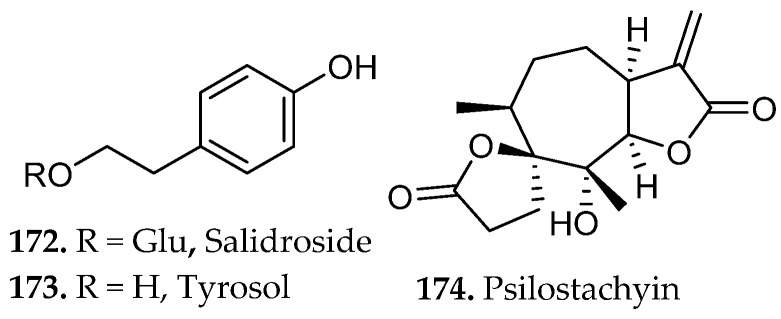
Natural products **172**–**174** extracted by green solvents.

NADES based on chloride combined with organic acids (malonic, malic, tartaric, or citric acid) were shown to be particularly effective for extracting glycosylated phenolic compounds from *Rhodiola rosea*, most notably salidroside (**172**, Figure 28), while also recovering its aglycone precursor tyrosol (**173**, Figure 28) [108]. Across all solvent systems evaluated, NADES consistently outperformed conventional hydroalcoholic solvents in the extraction of salidroside, reflecting the strong compatibility between highly polar, hydrogen-bond-rich NADES matrices and sugar-containing phenolics.

Notably, this enhanced solubility was less pronounced for aglycone species, suggesting that NADES composition preferentially stabilizes glycosylated derivatives, an effect attributed to the dense hydrogen-bonding network formed between choline chloride, organic acid NADES and the hydroxyl-rich glycosidic moieties. This selectivity became particularly apparent when comparing glycosylated phenolics to their less polar aglycone counterparts [108]. These findings establish NADES not only as green solvent alternatives but as inherently selective extraction media, with a structural bias toward glycosylated phenolics in *R. rosea* and meaningful implications for tailoring solvent systems to target compound class.

The primary challenge limiting the implementation of NADES is high viscosity, which restricts solvent penetration into plant matrices and reduces solute interactions. While viscosity can be reduced through heating or addition of water, these strategies risk degrading thermolabile compounds or altering selectivity. Continued optimization is needed to balance extraction efficiency with the inherent green chemistry advantages of NADES.

#### 6.1.3. Biobased Solvents

Bio-based solvents are derived from biomass, often produced through fermentation of lipids and sugars or steam distillation of organic materials. These solvents align with green chemistry principles, offering low volatility, reduced toxicity, biodegradability, and renewable sourcing. Common examples include ethanol, glycerol, limonene, and ethyl lactate.

Glycerol-based solvent systems demonstrated strong potential as green alternatives for polyphenol extraction from *Hesperethusa crenulata* (Thanaka) bark [109]. Glycerol/ethanol (1:1, *v*/*v*) extracts exhibited the highest total phenolic content and antioxidant capacity in ABTS and FRAP assays, while neat glycerol yielded the greatest overall compound diversity by LC–MS, outperforming ethanol and water alone.

Although aqueous extracts showed relatively strong DPPH scavenging activity, this effect was attributed to assay sensitivity toward hydrogen-donating species such as ascorbic and citric acids rather than superior polyphenol recovery. Overall, the enhanced extraction efficiency of glycerol-containing systems was linked to their tunable polarity, extensive hydrogen-bonding capacity, and low toxicity, supporting glycerol’s role as a biobased, sustainable solvent capable of replacing conventional organic solvents in phytochemical extraction workflows [109].

Similarly, MAE employing ethyl lactate, a biodegradable and bio-based solvent, afforded a higher overall extraction yield from *Ambrosia arborescens* than conventional methanolic maceration, while maintaining a comparable phytochemical profile [110]. Both extraction approaches yielded the same 28 secondary metabolites, with only two amino acid derivatives uniquely detected in the methanolic extract, indicating that replacement of methanol with ethyl lactate did not compromise chemical coverage. The characteristic sesquiterpene lactone psilostachyin (**174**, Figure 28) was recovered at similar levels by both methods, and antioxidant activities were comparable, demonstrating that biobased ethyl lactate can serve as an effective and sustainable alternative to petroleum-derived solvents in MAE without loss of extraction performance.

While bio-based solvents offer sustainable alternatives, the designation “bio-based” is not synonymous with “green”. Certain bio-derived solvents, including methanol and 2-methyltetrahydrofuran (2-MeTHF), retain undesirable properties such as flammability and toxicity. Therefore, each bio-based solvent requires individual assessment for safety, environmental impact, and extraction suitability for use as a green solvent.

### 6.2. Machine Learning and Artificial Intelligence Applications in Natural Product Research

Machine learning and AI are addressing critical bottlenecks in natural product discovery, including rediscovery of known compounds, inefficient prioritization, and trace metabolite isolation by predicting structural features from tandem mass spectra, accelerating dereplication, and prioritizing fractions before full isolation. Integrating spectroscopic, structural, and biosynthetic data, modern AI frameworks leverage molecular networking to identify novel scaffolds and chemically unusual metabolite families [111]. In microbial discovery, genome-mining tools paired with machine learning link biosynthetic gene clusters to predicted metabolite classes, streamlining identification of polyketides, nonribosomal peptides, terpenoids, alkaloids, and RiPPs [112,113].

Machine learning models, including neural networks and support vector regression, optimize extraction by modeling nonlinear variable relationships, matching or outperforming response surface methodology across ultrasound-, microwave-, and enzyme-assisted workflows [114,115]. Generative AI complements these efforts by expanding natural product-like chemical space in silico through recurrent neural networks and transformer-based scaffold generators [116]. Collectively, ML and AI are emerging as unifying frameworks across extraction optimization, metabolomics, dereplication, and scaffold generation, critically accelerating and de-risking the natural product discovery pipeline.

## 7. Separation, Purification, and Biological Activity Testing

Crude natural product extracts are chemically complex mixtures of primary metabolites (carbohydrates, lipids, proteins, inorganic salts) and structurally multifaceted secondary metabolites including alkaloids, terpenoids, flavonoids, saponins, steroids, and polyketide metabolites. This chemical diversity necessitates systematic fractionation and separation strategies to reduce complexity prior to structural characterization and biological evaluation. Following extraction, crude extracts undergo preliminary fractionation to partition metabolites by physicochemical properties before chromatographic purification. Liquid–liquid partitioning with immiscible solvent systems is the most widely employed approach, exploiting polarity and solubility differences to distribute metabolites across discrete phases. Sequential partitioning typically proceeds from nonpolar solvents, removing lipophilic constituents such as fatty acids, chlorophylls, and hydrocarbons through intermediate-polarity solvents enriching phenolics, terpenoids, and alkaloids, to highly polar fractions retaining glycosides, sugars, and organic acids. This stepwise process reduces chemical complexity within each fraction, improving the efficiency of downstream purification [117].

Solid-phase extraction (SPE) offers a complementary and highly versatile fractionation approach in which analytes are selectively retained on sorbent materials, including reversed-phase C18 silica, polyamide, Diaion HP-20, or ion-exchange resins, based on hydrophobic, polar, or electrostatic interactions. Elution with solvents of increasing polarity or ionic strength allows systematic recovery of compound classes with distinct physicochemical profiles, enabling efficient removal of interfering matrix components such as pigments, salts, and highly polar sugars prior to high-resolution chromatography [118].

Complementing these separation steps, early-stage analytical profiling is essential for characterizing extract composition and guiding purification strategy. Thin-layer chromatography (TLC) provides rapid, low-cost visualization of compound classes using selective spray reagents, while HPLC–UV offers quantitative resolution of UV-active constituents across complex mixtures. Most informatively, LC–MS coupling enables simultaneous chromatographic separation and mass spectral detection, allowing dereplication of known metabolites and prioritization of novel chemical entities based on molecular formula, fragmentation patterns, and database matching, substantially reducing redundant isolation efforts and focusing resources on chemically and biologically promising fractions [119].

Chromatographic techniques are the primary tools for resolving enriched fractions into discrete compounds. Open-column chromatography on silica gel or alumina remains widely used for polarity-based separations, exploiting differential adsorption and gradient elution to resolve compound classes. Normal-phase systems are particularly effective for nonpolar terpenes and moderately polar phenolics, while reversed-phase C18 chromatography has become the dominant platform for flavonoids, phenolic acids, glycosides, and other mid- to high-polarity metabolites, where separation is governed by hydrophobic interactions modulated through solvent gradient elution. Integration of ultraviolet-visible (UV–Vis), evaporative light-scattering, and mass spectrometric detection accelerates purification workflows and minimizes redundant isolation of previously reported compounds [118,119].

Structural characterization relies on complementary spectroscopic techniques to establish molecular identity. NMR spectroscopy remains the cornerstone of structure elucidation, with 1D (^1^H, ^13^C) and 2D experiments (COSY, HSQC, HMBC) defining atomic connectivity, functional groups, and stereochemistry. High-resolution MS and MS/MS provide accurate mass information and substructural clues that are especially useful for dereplication and feature prioritization in complex mixtures [119]. Increasingly, AI-augmented elucidation platforms complement conventional workflows through probabilistic candidate ranking, NMR parameter prediction, and automated annotation of complex datasets, which is particularly valuable when spectral overlap or conformational flexibility limits manual interpretation.

Additional spectroscopic methods provide critical supporting data for complete structural assignments. Infrared (IR) spectroscopy identifies characteristic functional groups through diagnostic absorption bands, while UV–Vis spectroscopy informs on chromophore systems and conjugation patterns relevant to phenolics, flavonoids, and polyenes. Optical rotation measurements and electronic circular dichroism (ECD) spectroscopy are indispensable for assigning absolute configuration, particularly for chiral-containing natural products lacking suitable crystalline forms [120]. When single crystals are obtainable, X-ray crystallography remains the definitive method for unambiguous three-dimensional structure determination, resolving both relative and absolute stereochemistry with atomic precision. The integrated application of these techniques alongside NMR and MS provides the comprehensive structural dataset required for reliable dereplication, confident assignment of novel scaffolds, and accurate reporting of stereochemical relationships essential for downstream biological evaluation.

Dereplication enables rapid identification of known compounds within crude extracts or partially purified fractions, allowing researchers to prioritize novel chemical entities and eliminate redundant isolation. Modern workflows rely on LC–MS and LC–HRMS platforms that compare accurate masses, isotopic distributions, and fragmentation spectra against curated natural product databases and spectral libraries [118]. High-resolution MS/MS generates diagnostic fragment ions revealing substructural features and functional group arrangements, enabling tentative structural assignment directly within complex mixtures without prior isolation [111].

In large-scale discovery programs, prefractionation coupled with LC–MS-guided dereplication enables rapid triaging of extract libraries ahead of full purification and bioassay evaluation, substantially reducing rediscovery rates and accelerating the identification of chemically unique metabolites [121].

**Figure 29:**
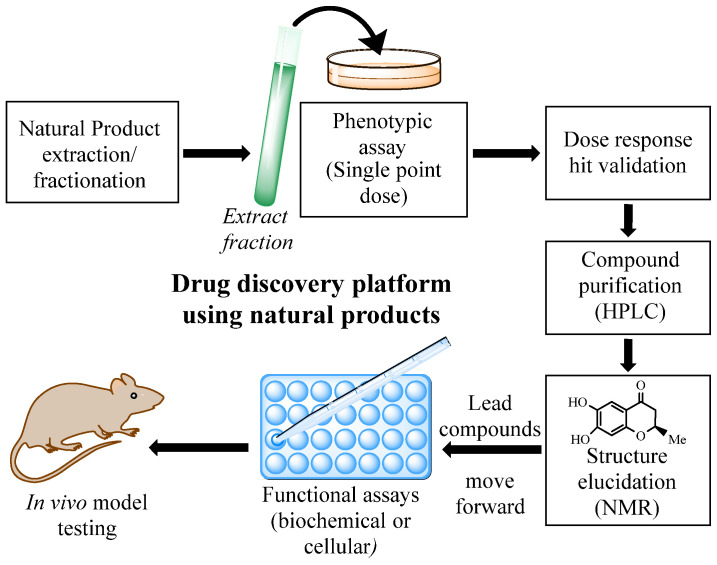
Schematic overview of a modern natural product drug discovery pipeline, illustrating the sequential workflow from initial dose–response screening through compound isolation and structural characterization to in vivo validation.

Following structural characterization and dereplication, biological evaluation determines the functional relevance of isolated metabolites and guides prioritization for further investigation as depicted in Figure 29. Initial screening employs targeted in vitro bioassays selected based on therapeutic application, commonly including antimicrobial susceptibility testing, cell viability assays (CTG luminescence or resazurin reduction) for cytotoxicity, and enzyme inhibition assays against biologically relevant targets. These assays function as an early activity filter, identifying bioactive constituents while minimizing unnecessary purification of inactive compounds. Modern discovery pipelines increasingly integrate high-throughput or semi-high throughput screening platforms, enabling rapid evaluation of large extract libraries and pre-fractionated samples while linking observed bioactivity to specific chemical constituents through parallel analytical profiling [43,121].

More recently, AI-supported informatics workflows integrate LC–MS/MS metabolomic profiling, feature-based molecular networking, and bioactivity-guided analysis enable correlation of mass spectral features with active fractions, recognition of structurally related metabolite families, and prioritization of chemically distinct or bioactive scaffolds for targeted isolation and downstream evaluation [43,122].

This integrated workflow encompasses separation, purification, structural elucidation, and biological evaluation, provides a systematic framework for advancing crude extracts toward actionable drug candidates. By combining AI-driven informatics, high-resolution chromatography, and multidimensional spectroscopic analysis, modern natural product discovery efficiently bridges chemical diversity and therapeutic potential, accelerating the identification of novel bioactive scaffolds with clinical relevance.

## 8. Extraction Approaches for Environmental Sustainability

Recent advances in extraction technology have introduced a range of innovative platforms aimed at recovering high-value constituents from complex natural matrices. These developments are driven by the growing demand for more targeted, scalable, and environmentally sustainable approaches to isolate and recycle valuable resources, including metals, minerals, and halogenated contaminants from natural and industrial sources. Metals and other carbon sources represent one of Earth’s most valuable and versatile natural resources, obtained either through traditional mining and extraction from ore deposits or increasingly through recycling and recovery processes. Primary extraction involves isolating metals such as iron, copper, lithium, and rare earth elements from the earth’s crust through mining, smelting, and chemical refining, processes that, while resource-intensive, remain essential for meeting global industrial demand [123,124,125,126].

Within the context of this review, extraction through recycling offers a compelling and sustainable alternative to recovering metals from end-of-life products, electronic waste, and industrial byproducts using hydrometallurgical and pyrometallurgical techniques. The societal benefits of this approach are substantial [124,125,126]. Recycling metals dramatically reduces energy consumption, producing recycled aluminum, for example, requires up to 95% less energy than primary production while simultaneously reducing greenhouse gas emissions, minimizing land disruption, and conserving finite ore reserves [127]. From an economic standpoint, a robust metal recycling infrastructure creates jobs, reduces dependence on geopolitically sensitive supply chains, and stabilizes the availability of critical materials essential for emerging technologies including electric vehicles, renewable energy systems, and medical devices.

A notable application of these emerging technologies is Flash Joule Heating (FJH), a conceptually innovative and technically compelling approach that leverages rapid electrothermal processing to selectively extract or sequester target components from complex matrices. By generating intense, ultrafast thermal pulses, FJH enables the efficient and selective capture of specific constituents, offering a powerful strategy for the recovery or remediation of valuable and harmful substances alike [127].

**Figure 30:**
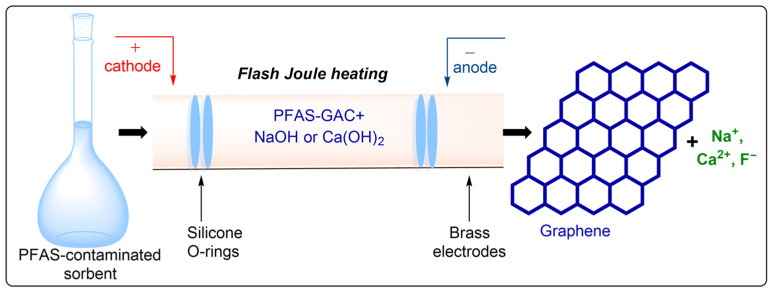
Flash joule heating method for extraction of perfluoroalkyl substances (PFAS).

The widespread contamination of freshwater systems by per- and polyfluorinated alkyl substances (PFAS), represents a significant and growing environmental challenge. These highly persistent compounds resist conventional degradation pathways, and while granular activated carbon (GAC) has been broadly adopted as a capture medium, the resulting PFAS-laden carbon waste remains notoriously difficult to manage safely and efficiently [127]. The selective extraction and remediation of persistent environmental contaminants from complex matrices have long represented a formidable challenge in environmental chemistry. Conventional approaches, while effective to varying degrees, are often hampered by high energy demands, the generation of hazardous secondary waste, and limited scalability. FJH leverages rapid, high-temperature processing in the presence of sodium or calcium salts to efficiently extract PFOA and PFOS from the carbon matrix and convert them into chemically inert fluoride salts. The application of flash Joule heating for the extraction of perfluoroalkyl substances is illustrated in Figure 30. The method achieves greater than 99% extraction efficiency for both PFOA and PFOS, with over 90% of the fluorine content successfully converted into stable, non-hazardous byproducts, all within approximately one second of processing time.

What sets this approach apart from existing technologies is its operational simplicity and sustainability profile. The process requires no solvents or catalysts, substantially reduces energy demands, and minimizes greenhouse gas emissions and secondary waste. Furthermore, the spent carbon is simultaneously upcycled into flash graphene, a high-value material that offsets remediation costs by an estimated $60–100 per kilogram [127]. Taken together, these attributes position FJH as a scalable, cost-effective, and environmentally responsible platform for PFAS extraction and carbon waste valorization.

Solvent extraction has emerged as an efficient and sustainable strategy for recovering and recycling critical metals from electronic waste, industrial effluents, and end-of-life products. These methods typically employ selective chelating agents, deep eutectic solvents (DESs), or ionic liquids (ILs) that preferentially coordinate target metal ions, enabling their separation from complex mixtures through liquid–liquid partitioning.

A compelling example is the recovery of lithium and other valuable metals including cobalt, nickel, and manganese from spent lithium-ion batteries (LIBs), a process critical to sustaining battery manufacturing supply chains and reducing dependence on primary mining operations. A recent study demonstrated the recovery of Co, Ni, Cu, Li, and Mn from the “black mass” of spent portable LIBs using choline chloride-based DESs combined with bifunctional ionic liquids, achieving up to 100% extraction efficiency for most target metals. Compared to conventional organic solvents, ILs and DESs offer notable advantages in terms of lower volatility, enhanced chemical and thermal stability, recyclability, and superior metal selectivity, while maintaining a reduced environmental footprint [128].

## 9. Conclusions

The extraction methodologies presented in this review illustrate how systematic manipulation of solvent polarity, temperature, pressure, and matrix composition controls the selective transfer of architecturally complex secondary metabolites from biological matrices into analytically compatible phases. Recent developments in both traditional and intensified extraction technologies enable researchers to optimize solvation environments for specific functional groups and molecular scaffolds, enhancing extraction efficiency, maintaining structural integrity, and facilitating subsequent bioactivity assessment.

Given that natural products continue to occupy chemical space with optimal drug-like properties, sophisticated isolation strategies are crucial for realizing their complete therapeutic potential. These advanced methodologies establish critical links between complex biological matrices and high-resolution structural elucidation and pharmacological evaluation, ensuring that natural products remain fundamental drivers of chemical discovery and lead compound identification in modern pharmaceutical research.

## Figures and Tables

**Table 1:** Types of deep eutectic solvents classified by HBD and HBA.

Type	Cat + X^−^ + Component	Examples
Type I	MCl_X_	M = Zn, Sn, Fe, Al, Ga, In
Type II	MCl_X_ · _n_H_2_O	M = Cr, Co, Cu, Ni, Fe
Type III	RZ	Z = CONH_2_, CO_2_H, OH
Type IV	MCl_X_ + RZ = MCl_X−1_^+^ · RZ + MCl_X−1_^−^	M = Al, Zn Z = CONH_2_, CO_2_H, OH

## Data Availability

No new data were created or analyzed in this study. Data sharing is not applicable to this article.
